# Stimuli‐Responsive Electrospun Fluorescent Fibers Augmented with Aggregation‐Induced Emission (AIE) for Smart Applications

**DOI:** 10.1002/advs.202204848

**Published:** 2022-11-14

**Authors:** Vishal Kachwal, Jin‐Chong Tan

**Affiliations:** ^1^ Multifunctional Materials & Composites (MMC) Laboratory Department of Engineering Science University of Oxford Parks Road Oxford OX1 3PJ UK

**Keywords:** aggregation‐induced emission (AIE), electrospinning, luminescence, mechanochromic, optoelectronics, photodynamics, sensors

## Abstract

This review addresses the latest advancements in the integration of aggregation‐induced emission (AIE) materials with polymer electrospinning, to accomplish fine‐scale electrospun fibers with tunable photophysical and photochemical properties. Micro‐ and nanoscale fibers augmented with AIE dyes (termed AIEgens) are bespoke composite systems that can overcome the limitation posed by aggregation‐caused quenching, a critical deficiency of conventional luminescent materials. This review comprises three parts. First, the reader is exposed to the basic concepts of AIE and the fundamental mechanisms underpinning the restriction of intermolecular motions. This is followed by an introduction to electrospinning techniques pertinent to AIE‐based fibers, and the core parameters for controlling fiber architecture and resultant properties. Second, exemplars are drawn from latest research to demonstrate how electrospun nanofibers and porous films incorporating modified AIEgens (especially tetraphenylethylene and triphenylamine derivatives) can yield enhanced photostability, photothermal properties, photoefficiency (quantum yield), and improved device sensitivity. Advanced applications are drawn from several promising sectors, encompassing optoelectronics, drug delivery and biology, chemosensors and mechanochromic sensors, and innovative photothermal devices, among others. Finally, the outstanding challenges together with potential opportunities in the nascent field of electrospun AIE‐active fibers are presented, for stimulating frontier research and explorations in this exciting field.

## Graphical abstract

This review gives an overview of the advanced composite fibers engineered from electrospinning of AIE‐active (aggregation‐induced emission) materials, for potential use in photodynamic and photothermal devices, optoelectronics, sensors, biology, and biomedicines. Detailed structure–property relationships are addressed in terms of AIE chemical structures, fiber morphology, and composite microstructures.

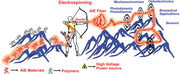

## 1 Background

Luminescence is defined as “the emission of electromagnetic radiation (light) from a material that does not result from heating.” The kind of energy that causes luminescence determines the distinct types, such as photoluminescence, cathodoluminescence, chemiluminescence, electroluminescence, mechanoluminescence, radioluminescence, and thermoluminescence. Photoluminescence is light emitted after light absorption. The name combines the Latin word luminescence and the Greek prefix photo‐ meaning light, thus photoluminescence is photon‐induced luminescence. Any photon absorption‐induced light emission is photoluminescence; however, chemists often sub‐divide it into “fluorescence” and “phosphorescence.” Fluorescence and phosphorescence can be differentiated on the basis of the quantum mechanics of the excited and ground states. Fluorescence may be described as photoluminescence in which the radiative transition does not require a change in spin multiplicity, whereas phosphorescence requires a change in spin multiplicity. Commonly, fluorescence and phosphorescence refer to photoluminescence observed in molecular systems.^[^ ^1^, ^2^ ^]^

It follows that photoluminescent (PL) materials radiate light without emitting heat energy, in response to light exposure at another wavelength.^[^ ^3^ ^]^ Generally, the excitation source is an UV light with a shorter wavelength of about 300−400 nm, while the visible light being emitted has a longer wavelength in the range of 400−700 nm. Solid‐state PL materials, encompassing inorganic or organic compounds, are central to numerous technological applications.^[^ ^4^, ^5^, ^6^, ^7^, ^8^ ^]^ Conventional PL materials have been extensively studied and developed for decades given their applications in various fields, such as light‐emitting diodes (LEDs), optoelectronics, chemical and biological sensing, fluorescent dyes, and imaging.^[^ ^9^, ^10^ ^]^

**Figure 1:**
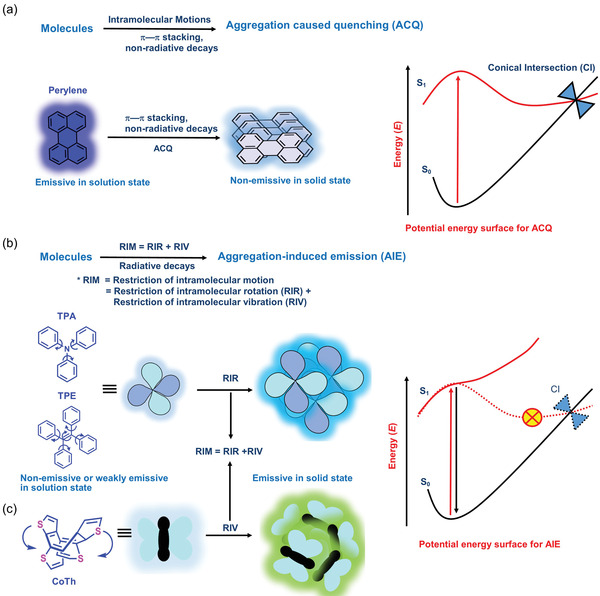
Basic concepts of aggregation‐induced emission (AIE) versus aggregation caused quenching (ACQ). a) Source of ACQ in conventional fluorescent materials, such as perylene (C_20_H_12_) that quenches in a solid‐state aggregate. b) Mechanisms for AIE ascribed to the restriction of intramolecular motion (RIM), including the restriction of intramolecular rotation (RIR) of a propeller‐shaped molecule (most common examples are TPA and TPE). Adapted and redesigned under the terms of the CC‐BY creative Common Attribution 4.0 International License.^[^ ^21^ ^]^ Copyright 2019, Oxford Academic and c) the RIV of a butterfly‐shape structure (e.g., cycloocta‐tetrathiophene (CoTh) molecule) in aggregated state. Adapted and redesigned under the terms of the CC‐BY creative Common Attribution 4.0 International License.^[^ ^22^ ^]^ Copyright 2019, Springer Nature. The panels on the right illustrate the potential energy surfaces of the aggregated states, showing the cases with and without a conical intersection (CI) for ACQ and AIE, respectively. S_0_ and S_1_ are the ground and excited states, respectively.

**Figure 2:**
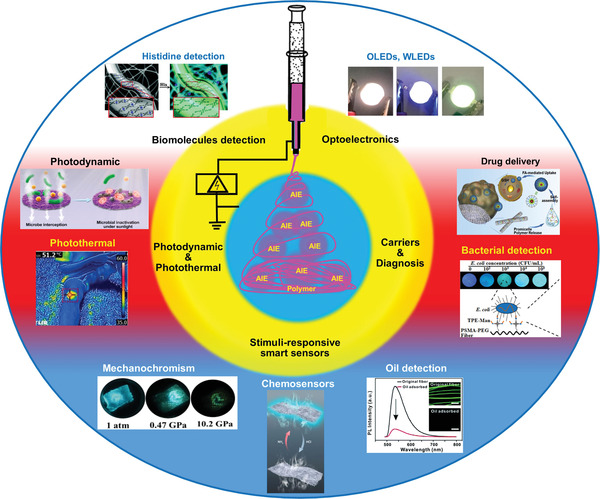
Emerging applications of luminescent AIE‐active polymeric fibers constructed by electrospinning for deployment in multiple sectors, encompassing optoelectronics—OLEDs and WLEDs, , drug carriers, bacterial detection, and killing, fluorescent bioprobes, stimuli‐responsive fluorescent fibers as smart sensors for detection of pressure, oil, and chemical vapors, photothermal and photodynamic conversions. Image of OLEDs, WLEDs: Reproduced with permission.^[^ ^23^ ^]^ Copyright 2022, Wiley‐VCH. Image of drug delivery: Reproduced with permission.^[^ ^24^ ^]^ Copyright 2018, Elsevier. Image of bacterial detection: Reproduced with permission.^[^ ^25^ ^]^ Copyright 2015, American Chemical Society. Image of histidine detection: : Reproduced with permission.^[^ ^26^ ^]^ Copyright 2015, Royal Society of Chemistry. Image of mechanochromism: Reproduced with permission.^[^ ^27^ ^]^ Copyright 2020, Wiely‐VCH. Image of oil detection: Reproduced with permission.^[^ ^28^ ^]^ Copyright 2014. Image of chemosensor: Reproduced with permission.^[^ ^29^ ^]^ Copyright 2021, American Chemical Society. Image of photothermal: Reproduced with permission.^[^ ^31^ ^]^ Copyright 2020, Wiely‐VCH. Image of photodynamic: Reproduced with permission.^[^ ^30^ ^]^ Copyright 2021, Elsevier.

Because of the strong intermolecular *π*−*π* stacking effect, most PL materials are nonemissive in the solid‐state or in a concentrated solution state, where fluorescent molecules (fluorophores) become aggregated. This phenomenon is called “aggregation‐caused quenching” (ACQ), as elucidated in **Figure** **1**a for the case of a perylene aggregate. The prevalence of ACQ has imposed constraints on the applicability of conventional PL materials for constructing miniaturized sensors and tunable devices.^[^ ^11^, ^12^, ^13^ ^]^ To address this limitation, several strategies have been proposed, including guest–host confinement by utilizing nanoporous frameworks,^[^ ^14^, ^15^ ^]^ core–shell structures,^[^ ^16^ ^]^ co‐assembly of chromophores and molecular barriers,^[^ ^17^ ^]^ and aggregation‐induced emission (AIE).^[^ ^18^, ^19^, ^20^ ^]^ In this review, we will concentrate on the concepts of AIE (Figure 1b,c) in conjunction with electrospinning methodologies to accomplish high‐efficiency fluorescent fibers unimpeded by ACQ. There are numerous potential applications for leveraging novel AIE‐active electrospun fibers, porous mats, membranes, and thin‐film nanocomposites, some of which are summarized in **Figure** **2**.

### 1.1 Aggregation‐Induced Emission (AIE)

In 2001, as opposed to ACQ, a revolutionary new concept, termed AIE, was first introduced by Tang and co‐workers.^[^ ^32^ ^]^ Remarkably, it was demonstrated that bulky rotor‐propeller‐shaped molecules that are nonemissive in the diluted state become highly emissive in the aggregated or concentrated states. Figure 1b,c illustrates this counterintuitive AIE phenomenon. AIE materials, also known as AIE luminogens or, in short, “AIEgens,” present several advantages over conventional PL materials, such as high quantum yield, low photobleaching, and no detrimental ACQ effects. Intermolecular and intramolecular interactions in molecular packing play a key role in the generation of AIE properties.^[^ ^18^, ^20^, ^33^ ^]^ The AIE concept has since expanded into many research fields, and the multifaceted research surrounding AIE materials has continuously grown and expanded into the cognate disciplines.^[^ ^34^, ^35^, ^36^ ^]^

Tetraphenylethylene (TPE) and triphenylamine (TPA) and their derivatives, are two of the most common AIE‐active materials in which the intramolecular motion of the phenyl rings is restricted in the aggregated state, resulting in enhanced radiative decay. Restricted intramolecular motion (RIM) has been accepted as a well‐known mechanism responsible for the AIE effect, as depicted in Figure 1b for the TPA and TPE aggregates.^[^ ^37^ ^]^ Further to RIM mechanism, the J‐type aggregation,^[^ ^38^ ^]^ restriction of intramolecular motions of the twisted structure,^[^ ^39^ ^]^ suppression of Kasha's rule,^[^ ^40^, ^41^ ^]^ and excited‐state intramolecular proton transfer (ESIPT)^[^ ^42^ ^]^ are other general mechanisms proposed for the AIE phenomena.^[^ ^43^ ^]^

Tang et al. in 2020 briefly described the various forms of RIM mechanism through different models.^[^ ^21^ ^]^ The internal conversion caused by the S_1_−S_0_ vibronic coupling is high‐speed for the AIE system with active molecular motions, thus resulting in a low PL emission. Due to the restriction of intramolecular vibrations (RIV) in an aggregate (Figure 1c), the PL emission of AIEgens significantly increases. AIE‐active molecules with many bulky rotor groups in the excited state undergo flexible molecular motions and experience rapid relaxation to a conical intersection (CI),^[^ ^44^ ^]^ see the potential energy curves depicted in Figure 1. The CI is the degenerate state of S_1_ and S_0_ where the magnitude of the vibronic interaction approaches infinity, causing a nonradiative decay. In the aggregated state, the emission intensity increases by restricting access to the conical intersection due to the hindrance of molecular motion.^[^ ^45^ ^]^ Some of the excited states (such as *n*, π* state/charge transfer state/symmetry forbidden transition) of AIEgens are considered a dark state compared to the (*π*, π* state)/locally excited (LE)/symmetry‐allowed transition states. Because of the low transition probability attributed to the small molar absorptivity and oscillator strength, the nonradiative decay constant of the aggregates of AIEgens becomes dominant relative to the radiative decay constant, thus *K*_nr_ ≫ *K*_r_. In aggregated states, the restriction of molecular motion prevents access to the dark state, which hinders emission recovery. Moreover, the AIEgens can undergo a photochemical reaction in the excited state, such as photoisomerization, photocyclization, and photoionization, which further increases nonradiative decay. For such AIE molecules, the photochemical reaction is suppressed by the restriction of molecular motion in the aggregated state.^[^ ^21^ ^]^

Since the first discovery of AIE materials just over two decades ago, the AIE‐based research has swiftly expanded into several subfields of study. Today, AIE systems are diverse, encompassing CIE: crystallization‐induced emission; RTP: room‐temperature phosphorescence; aggregation‐induced delayed fluorescence; anti‐Kasha transition; clusterization‐triggered emission; through‐space interaction; ML: mechanoluminescence; circularly polarized luminescence; AIG‐ROS: aggregation‐induced generation of reactive oxygen species; photothermal/photoacoustic phenomena; solid‐state molecular motion; polymerization‐induced emission; matrix coordination‐induced emission.^[^ ^43^, ^46^, ^47^, ^48^, ^49^, ^50^, ^51^ ^]^ For heaving metal sensing with AIEgens, the relevant mechanisms encompass chelation‐assisted rigidification, cleavage‐triggered aggregation, metal‐bridged functionalization, and coordination‐induced complexation.^[^ ^52^, ^53^, ^54^ ^]^ Interested readers may acquire further details for each subfield through a number of reviews dedicated to AIE materials referenced above. AIEgens are an emerging area that exhibits remarkable photodynamic, photoacoustic, and photothermal therapeutic efficacy. Using a simple and innovative method, AIEgens may hasten the development of unconventional theranostic platforms. Moreover, AIEgens may be developed to detect enzymes crucial for biology and medical studies by leveraging the RIM mechanism.^[^ ^55^, ^56^, ^57^ ^]^

While there is a profound understanding of the PL mechanisms underpinning the photophysical properties of conventional fluorescent materials, the same cannot be said about the AIE materials. Notably, not only that the methods for synthesizing AIE‐active molecules are constantly evolving, but the basic understanding of the core processes^[^ ^50^, ^58^ ^]^ is equally expanding for establishing structure–property relationships to enable the rational design, fabrication, and cutting‐edge applications of multifunctional AIE systems (Figure 2).

### 1.2 Electrospinning of Thin Fibers

Electrospinning, an electrostatic fiber production technology, has gained popularity over the past decade due to its versatility and many potential applications.^[^ ^59^ ^]^ For example, electrospun fibers can be used in tissue engineering, protective clothing, filtration and distillation, rechargeable batteries, optoelectronics, environmental engineering, biosensors, wound dressings, and drug administration. Fine‐scale polymer fibers with diameters ranging from ≈10 nm to 5 µm can be fabricated by electrospinning routes, which employ electrical forces to produce “thin” fibers from polymer solutions of natural and synthetic polymers. More precisely, nanofibers can be defined as fibers whose diameter is less than ≈1 µm, which are typically in the range of 10s to 100s nm. Though the majority of AIE‐based fibers reported are in fact with a nominal diameter of 1 µm or so, strictly speaking these are “microfibers.” Fewer exemplars^[^ ^27^, ^29^ ^]^ involve AIE‐based electrospun “nanofibers” of less than 1 µm in diameter. Nanofibers have the advantage of having a greater surface‐to‐volume ratio, but will be mechanically weaker than microfibers in terms of their load‐bearing capacity.

**Figure 3:**
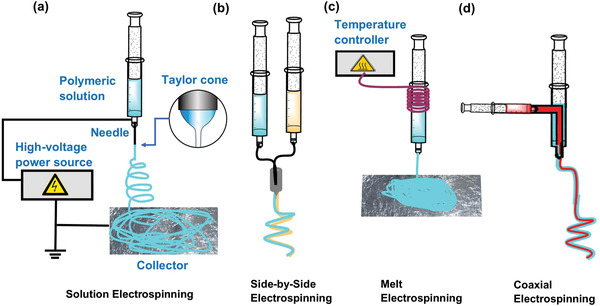
Schematics of the main electrospinning techniques used to prepare polymer fibers that incorporate AIEgens. a) Basic electrospinning setup; inset shows the geometry of a typical Taylor cone, b) side‐by‐side (Janus) electrospinning, c) melt electrospinning with temperature control, and d) coaxial electrospinning.

The basic idea underpinning electrospinning can be traced back to 1882, when Lord Rayleigh wrote an article about the instability of thin liquid jets subjected to an electric field.^[^ ^60^ ^]^ Electrospraying was a method for electrically spreading fluids patented by Cooley^[^ ^61^ ^]^ and Morton^[^ ^62^ ^]^ in 1902. The essential idea of electrospraying was the same as that of electrospinning, which was patented by Formhals^[^ ^63^, ^64^ ^]^ in 1934 and 1944. While electrospraying uses a low‐molecular‐weight solution, electrospinning requires a high‐molecular‐weight polymer solution to yield extended fibers. In 1960s, Taylor demonstrated how to mathematically describe and simulate the spherical to conical shape shift of a polymer solution or melt droplet under the effect of a strong electric field (hence called a Taylor cone).^[^ ^65^, ^66^ ^]^ Electrospinning is an electrohydrodynamic technique zin which a liquid droplet is electrified to create a jet, which is then stretched and elongated to create thin fibers, as illustrated in **Figure** **3**. The basic setup for electrospinning is relatively straightforward, as depicted in Figure 3a. In essence, it consists of a high‐voltage power source, a syringe pump, a spinneret (e.g., nozzle of a blunt hypodermic needle), and a conductive collector. The power source can be either DC (direct current) or AC (alternating current). Due to surface tension, the liquid polymer is ejected from the spinneret during electrospinning. Electrified electrostatic repulsion between the same‐sign surface charges deforms the droplet into a Taylor cone, from which a charged jet is emitted, see inset of Figure 3a.^[^ ^67^, ^68^ ^]^

Although the jet initially stretches straight, it then whips due to bending instabilities. As the jet is extended into finer diameters, it hardens, depositing solid fibers onto the collector. The precise geometry of the collector can be designed to accomplish different fiber network architectures. For example, a dense metal collector (e.g., aluminum foil) will generate a randomly oriented fibrous membrane, whereas a porous collector (e.g., copper mesh) will yield a patterned morphology via electrostatic lensing.^[^ ^69^ ^]^ Many reviews comprehensively overview the electrospinning process, including the basic principles, techniques, materials, and applications.^[^ ^70^, ^71^ ^]^

Electrospinning can produce thin fibers from various materials, especially organic polymers in the form of a solution or melt. Small molecules can also be electrospun into fibers if they self‐assemble and yield sufficient chain entanglement or if they dissolve in conductive polymers at the optimum concentration to form electrospun fibers. Electrospinning is commonly performed in the far‐field mode, with a hollow needle serving as the spinneret and a metal substrate acting as the collector.^[^ ^72^ ^]^ The laboratory usually prefers this method to produce small sample quantities or optimize electrospinning conditions. Multiple parameters like the distance between tip of spinneret to the collector, the number of nozzles, and the configuration of the spinneret can be adapted to obtain the desired size and shape of fibers.

The single‐needle configuration (Figure 3a) is most frequently used for producing nanofibers in small quantities in a laboratory setting.^[^ ^71^ ^]^ The other electrospinning techniques are side‐by‐side electrospinning, melt electrospinning, blend electrospinning, coaxial electrospinning, and emulsion electrospinning. Side‐by‐side electrospinning is also called the Janus electrospinning process as shown in Figure 3b, where one can use different polymeric solutions with the help of multiple nozzles for electrospinning simultaneously.^[^ ^73^ ^]^ The solvent‐free fibers can be made through melt electrospinning depicted in Figure 3c, where the polymer is passed through a high‐temperature coil before going to the spinneret.^[^ ^74^, ^75^ ^]^ Melt electrospinning enables medical‐grade polymers to be treated “as received” from the supplier, thus avoiding the addition of toxic solvents. Moreover, insoluble polymers in solvents can be processed by melt electrospinning. In blend electrospinning, drugs or medicine are first combined with the polymeric solution before the electrospinning process is performed.^[^ ^76^ ^]^

Two or three polymeric liquids are electrospun by a specially designed spinneret in coaxial electrospinning, see Figure 3d. Core/shell electrospinning uses two coaxial needles. The spinneret forms composite polymeric droplets by pumping the core liquid through the inner needle and the shell material through the outer needle. Strong electric fields generate composite electrospinning jets and core/shell fibers from polymeric droplets. The main requirement for the coaxial‐type electrospinning is that the shell polymer should be an electrospinnable solution, while the core polymeric solution could be a nonspinnable liquid. Coaxial electrospinning may be used to create fibrous meshes from nonspinnable or scarcely spinnable materials. Coaxial electrospinning is mainly used for fabricating fibers targeting drug delivery purposes.^[^ ^77^, ^78^ ^]^ In contrast, emulsion electrospinning is based on the single‐nozzle formation of the core–shell structure. Emulsion electrospinning is the combination of both blend and coaxial electrospinning. Electrospinning of stable emulsion of two or more liquids creates core–shell fibers without the need for introducing two coaxial needles. The electrospun solution is based on the separated liquid phases, where the continuous phase forms fiber shells while the droplet phase forms the fiber cores.^[^ ^79^, ^80^ ^]^ The other kind of electrospinning method is where the nanoparticle meets the fiber; electrospinning may produce high stability and large‐scale organized nanoparticles, opening up a new pathway for the production of diverse multifunctional materials.^[^ ^81^, ^82^ ^]^

Notably, the polymeric solutions are elongated and stretched in the electric field in the electrospinning process, thereby enhancing the molecular orientation and alignment.^[^ ^83^, ^84^ ^]^ Due to the higher alignment and orientation, the intermolecular *π*−*π* interaction and energy transfer between the molecules are effectively restricted, thereby reducing the nonradiative decay and increasing the fluorescence intensity of resultant PL materials.^[^ ^85^, ^86^, ^87^, ^88^ ^]^ Stimuli‐responsive luminescent electrospun fibers can be exploited for self‐cleaning (temperature‐sensitive) surfaces, in targeted drug delivery, and pH‐sensitive materials for medium monitoring.^[^ ^89^, ^90^, ^91^ ^]^ Ding et al. compared the properties of electrospun nanofibers with thin‐film coating as fluorescent probes and concluded that there is no ideal morphology for the best result. Each material is the best PL sensor in its specific environment.^[^ ^92^ ^]^ Optically active nanofiber depends upon the composition, structure, and material synthesis. The PL electrospun fiber can be fabricated by introducing the conjugated luminescent polymers or embedding fluorophores into the optically inert polymers.^[^ ^13^, ^88^ ^]^ Another approach for preparing PL electrospun nanofiber is by modifying the surface of the polymers by attaching chromophores through chemical functionalization.^[^ ^93^, ^94^, ^95^ ^]^ AIE materials are superior to other materials for making luminescent thin films due to their aggregate photo efficiency and improved photostability, which are the critical parameters for practical applications in sensors and optoelectronics devices.^[^ ^46^, ^96^ ^]^

Research on the utilization of AIE materials for fabricating electrospun nanofibers is fast gathering momentum. Recently, three reviews have been published that focused on the improved optical performance of AIEgens blended with nanofibers, and AIE polymers design to afford a range of biological and sensing applications. These work demonstrate the diversity of the optical performance and functionalities of AIE when paired with electrospun fibers.^[^ ^97^, ^98^ ^]^ The recent reviews have covered the various excited state phenomena associated with AIEgens, such as TICT (twisted intramolecular charge transfer), PET (photo‐induced electron transfer), RIR (restriction of intramolecular motion), and ISC (intersystem crossing) in fiber, which have been used for smart sensing in fields such as biomedical, energy conversion, and electronics.^[^ ^98^, ^99^ ^]^ Existing reviews concentrate on how AIE properties are enhanced in the fiber; however, the relationship between fiber morphology and structural interaction with AIE aggregates is not thoroughly explored. Until now, the attention was given to the incorporation of AIE materials in fiber and the plausible AIE mechanisms.^[^ ^97^, ^98^, ^99^ ^]^ Encouragingly, the results to date suggest that electrospun nanofibers and porous films/membranes incorporating AIEgens hold a lot of promise, since they offer enhanced photostability, photothermal properties, photoefficiency, and sensitivity. In this review, we have adopted a different approach and concentrate our discussions on the structure−processing−property relationships of AIE‐active electrospun fibers. An attempt is made to describe the qualities and applications of AIE‐electrospun fiber in terms of fiber morphology and the underpinning microstructures. Significant findings from recent exemplars are critically analyzed, comprising not only small AIE molecules and their derivatives but also macromolecular systems. Our objective is to gain a deeper insight into the role of electrospun fibers environment and the AIE–polymer interactions. To this end, special attention is given to understanding the combined effects of chemical structures and electrospinning pathways have on the fiber architectures and the resultant stimuli‐responsive behavior of a composite system. The current review will elucidate how the electrospun fiber provides a tunable micro/nanoscale environment for controlling the aggregation mechanism in an AIE–polymer assembly, bestowing unique functions not found in either AIEgen (filler) or polymer (matrix) treated in isolation. This review will pave the way to the design and engineering of efficient composites based on AIE‐electrospun nanofibers for fundamental and applied research.

## 2 AIE Electrospun Nanofiber Composites

### 2.1 Photoefficient AIE‐Active Fibers for Optoelectronics

Light‐emitting electrospun nanofibers are an emergent class of materials with tailorable mechanical properties, increased surface‐to‐volume ratio, anisotropic optoelectronic properties, and enhanced luminescence performance compared with conventional solution‐processed thin films.^[^ ^88^, ^100^ ^]^ The multicomponent nature of the luminescent nanofibers can yield different functionalities ideal for the fabrication of optoelectronic devices,^[^ ^101^ ^]^ such as LEDs,^[^ ^100^, ^102^ ^]^ photovoltaics and field‐effect transistors,^[^ ^103^, ^104^ ^]^ tunable waveguides and photonic sensors.^[^ ^105^, ^106^ ^]^ By incorporating AIE‐active materials into a polymer matrix to engineer bespoke nanofiber composites, this methodology paves the way to the rational design and synthesis of photoefficient solid‐state light emitters. More precisely, during electrospinning, the polymer solution undergoes mechanical elongation along the fiber axis, this axial strain results in the alignment of transition dipoles that alter the emission and absorption properties.^[^ ^107^, ^108^ ^]^ It has been proposed that due to the elongation and alignment of the polymeric chains in the electrospun fiber, the molecular motion of AIEgens becomes hindered, thus increasing the emission intensity.

**Figure 4:**
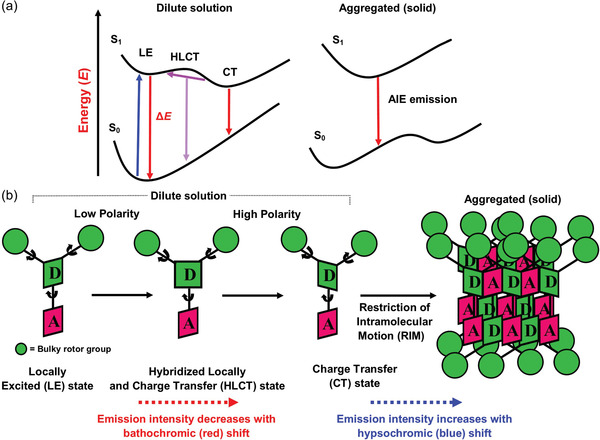
Donor (D)−acceptor (A) type of AIEgens. a) Potential energy surfaces show the possible transition states in dilute solutions compared with the solid‐state aggregates. b) Left: LE, HLCT, and CT state in dilute solution as a function of polarity, causing a red shift. Right: RIM in aggregated solid, resulting in a blue shift.

Heteroatom‐containing AIE‐based fluorescent materials (AIEgens) have substantial emission and electroactive properties.^[^ ^109^, ^110^ ^]^ The potential energy diagrams in **Figure** **4**a show that by introducing the “donor” (D) and “acceptor” (A) groups as a D−A pair or D−*π*(linker/spacer)−A confers a push–pull effect. This strategy is commonly adopted to tune the optical bandgaps of AIEgens.^[^ ^111^ ^]^ Most D−A types of AIEgens consist of different transition states, i.e., the locally excited (LE) state, charge transfer (CT) state, and hybridized local and charge transfer state (HLCT), as depicted in Figure 4. In a nonpolar environment, the fluorophore is LE and emits strongly. In a polar environment, intramolecular rotations can switch the fluorophore from the LE to CT state, this is accompanied by a reduction of energy gap as shown in Figure 4a. The HLCT state exists, 1) if the excited state has a significant transition moment from the LE state, and 2) if there is a total utilization of the exciton from the CT state.^[^ ^112^, ^113^, ^114^ ^]^ As the fluorophore experiences a significant change in shape, the emissions of the CT state is progressively red‐shifted with a higher solvent polarity (Figure 4b). AIEgens with twisted intramolecular charge transfer (TICT) characteristics may undergo the RIM process, enhancing the emission efficiency.^[^ ^115^, ^116^ ^]^ The D−A type AIEgens exhibit an excellent combination of high quantum efficiency, low photobleaching, and improved resilience to the surrounding environment.^[^ ^117^ ^]^

**Figure 5:**
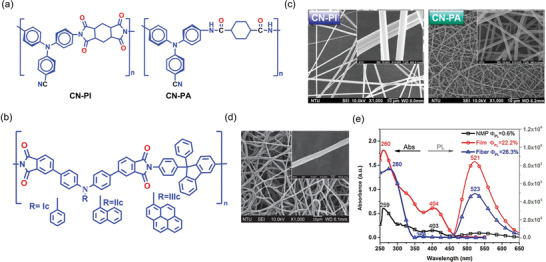
a,b) Chemical structures of AIE‐active polymers for electrospinning of nanofibers. c,d) FESEM images of nanofibers of CN‐PI, CN‐PA, and IIIc, without any bead formation. c) Reproduced with permission.^[^ ^119^ ^]^ Copyright 2013, Royal Society of Chemistry. e) Absorbance and PL emission spectra of IIIc in NMP solution, as solid film, and as electrospun fibers. Φ_PL_ denotes PL quantum yield. d,e) Adapted with permission.^[^ ^123^ ^]^ Copyright 2013, Wiley‐VCH.

Motivated by the propeller‐shaped configuration and electron‐donating behavior of TPA,^[^ ^118^ ^]^ Yen et al. in 2013, fabricated two TPA‐derivative‐loaded electrospun nanofibers (namely, CN‐PI and CN‐PA) with enhanced luminescence compared with the pristine AIE material.^[^ ^119^ ^]^ By leveraging the cyano (CN) functional group, the embedding of CN‐TPA into the polyimide (PI) or polyamide (PA) monomers resulted in the chemical structures shown in **Figure** **5**a. TPA is well known for its high PL and electroactive properties.^[^ ^120^ ^]^ The resulting polymers: CN‐PI and CN‐PA are both AIE active, having a photoluminescence quantum yield (PLQY) of 34% and 14% in the solution state, while the PLQY of the solid film risen to 65% and 46%, respectively. When the CN‐PI and CNPA polymers were fabricated into nanofibers by electrospinning (Figure 5c), there was a bathochromic (red) shift in the absorption spectra of the fiber accompanied by a higher PLQY (70% and 57%, respectively) compared with the solution‐ and solid‐state measurements, see Figure 5e. The bathochromic shift indicates the preferred orientation and a greater alignment of the polymeric chains along the fiber axis caused by electrospinning.^[^ ^119^ ^]^ Wholly aromatic polyimides are superior in their thermal and chemical stability, flame resistance, radiation resistance, mechanical strength, and flexibility compared to other polyimides.^[^ ^121^, ^122^ ^]^ AIE‐active wholly aromatic triarylamine‐based polyimides have been fabricated by the same group, designated as Ic, IIc, and IIIc (Figure 5b), yielding electrospun nanofibers with high‐efficiency photoluminescence. The electron microscopy images of the AIE‐active nanofibers show a smooth surface morphology, without any bead formation (Figure 5c,d). The PL spectra also exhibit a bathochromic shift compared to its solid film, indicating a more significant orientation and alignment of the polymer chains in the fiber form. The PLQY of fiber also rises to 26% compared to its film at 22% (Figure 5e).^[^ ^123^ ^]^

**Figure 6:**
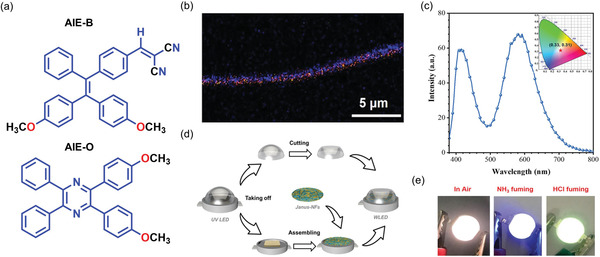
a) Chemical structures of AIE‐B and AIE‐O. b) Fluorescence image of Janus‐NF fabricated by side‐by‐side electrospinning, doped with AIE‐B and AIE‐O on the opposite sides of the fiber. c) Emission spectrum of Janus‐NFs and its corresponding CIE 1931 chromaticity diagram (inset), under UV 359 nm excitation. d) WLED device fabrication incorporating Janus‐NFs, and e) WLED testing in air, after ammonia fuming, and in an HCl environment. Adapted with permission.^[^ ^23^ ^]^ Copyright 2022, Wiley‐VCH.

Qin et al.^[^ ^124^ ^]^ devised a method for producing a flexible white light‐emitting nanofiber by altering the mass ratios of blue and orange emissive AIE molecules in polymers. The procedure is time‐consuming, and there is a risk of energy transfer when combining two solutions prior to electrospinning. To overcome such difficulty, Zhao et al.^[^ ^23^ ^]^ employed a side‐by‐side electrospinning method (Figure 3b) to create flexible white light emissive Janus nanofiber membranes (Janus‐NFs), by loading stimuli‐sensitive AIEgens (namely, AIE‐B and AIE‐O) (**Figure** **6**a) with complementary blue and orange emissions into a thermoplastic polyurethane (PU) polymer. The confocal laser scanning microscopy (CLSM) image (Figure 6b) shows that AIE‐B is found predominantly on the top side of the Janus fiber, whereas AIE‐O is present on the opposite side. Slow evaporation promotes partial diffusion of AIEgens prior to nanofiber solidification, resulting in the formation of an intriguing blue‐orange emitting interface due to color mixing at the diffusion layer. Because of rapid solidification, most AIEgens were scattered on either side of the outer surfaces of the Janus‐NFs. As a result, the distance between the two AIEgens was large enough to inhibit energy transfer. Janus‐NFs comprising 1% AIE‐B and 0.33% AIE‐O have a high quantum yield of ≈65%, with CIE coordinates (0.33, 0.31) close to the ideal white light emission (Figure 6c). Subsequently, a white light‐emitting device (WLED) was constructed by connecting Janus‐NFs to a GaN (gallium nitride) LED chip as UV source. The resultant WLED assembly emits a bright white light as shown in Figure 6d. However, when exposed to ammonia vapor, the emission color of the WLED switched from white to blue, and from white to green when exposed to HCl vapor (Figure 6e).^[^ ^23^ ^]^ This study demonstrated a unique concept for producing a photoefficient stimuli‐sensitive WLEDs by integrating AIEgens.

Of note, the propeller‐shaped bulky rotor with a D‐A type structure (Figure 4) would be an appealing choice for electrospinning to create AIE‐electrospun nanofiber with improved photoefficiency and quantum yield. Polymeric nanofibers become highly elongated and stretched out during the electrospinning process, thereby resulting in nanoconfined regions. The limitation of AIEgen mobility inside the fiber causes more significant radiative decay, which enhances the quantum yield. Further examples of this beneficial effect will be presented in sections below.

### 2.2 AIE Electrospun Fibers for Oil Adsorption

**Figure 7:**
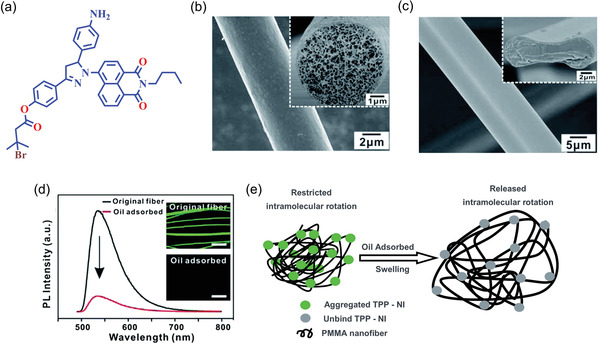
a) Chemical structure of TPP‐NI. b,c) Nanoporous and dense fibers electrospun in DMF and THF solvents, respectively. d) Photoluminescence quenching, the insets show the reduction of the fluorescence of TPP‐NI porous fiber before and after silicone oil adsorption. e) Plausible fluorescence quenching mechanism of TPP‐NI porous fibers upon oil adsorption. (Abbreviation: TPP‐NI ‐ 4‐(5‐(4‐aminophenyl)‐1‐(2‐butyl‐1,3‐dioxo‐2,3‐dihydro‐1H‐benzo[de]isoquinolin‐6‐yl)‐4,5‐dihydro‐1H‐pyrazol‐3‐yl)phenyl 3‐bromo‐3‐methylbutanoate). Adapted with permission.^[^ ^28^ ^]^ Copyright 2014, Royal Society of Chemistry.

Electrospun fibers with high porosity and superhydrophobicity are useful for use in oil recovery, waste water treatment, and protective clothing.^[^ ^125^, ^126^, ^127^ ^]^ To study the oil adsorption and desorption mechanism, Yuan et al.^[^ ^28^ ^]^ developed an AIE‐active nanoporous fiber comprising a modified polymethyl methacrylate (PMMA), incorporating TPP‐NI whose chemical structure is shown in **Figure** **7**a. The electrospun fiber was fabricated in *N*,*N*‐dimethylformamide (DMF) solvent with a polymer weight ratio of 30 wt% for porous structure (surface area ≈40 m^2^ g^−1^), and in tetrahydrofuran (THF) solvent with a polymer weight ratio of 25 wt% for solid fiber (surface area ≈0.4 m^2^ g^−1^), as shown in Figure 7b,c. The average diameter of the nanoporous electrospun fibers was found to be ≈4.25 µm, they emit a yellowish‐green fluorescence as shown in Figure 7d inset.

The hydrophobicity of the AIE‐active PMMA fibers was confirmed by contact angle measurement of 135°. The fluorescence intensity decreases drastically when the nanoporous fiber adsorbs silicone oil (see Figure 7d). This fluorescence quenching phenomenon can be explained by the swelling of the fiber upon oil uptake (radial enlargement > 20%), thereby creating free volume for intramolecular rotations of the AIE moieties, see Figure 7e. On expansion, the intramolecular rotations increase nonradiative decay, because the absorbed photonic energy is converted into kinetic energy via rotations and vibrations of AIEgens. Furthermore, the AIE‐active PMMA fiber was tested five times to remove oil from a mixture of water and oil, demonstrating the reusability of the porous membrane as an added advantage for practical application.^[^ ^28^ ^]^

The foregoing results revealed that nanoporous electrospun fibers and membranes are effective for oil adsorption and desorption. They may accomplish bespoke functionalities such as turn‐on/‐off sensing or ratiometric fluorescence when augmented with AIE materials.^[^ ^14^ ^]^

### 2.3 AIE Electrospun Nanofibers for Biological Applications

Fluorescent bioprobes have numerous potential in the biological sciences, biomedicine, and biotechnology, as they offer direct visualization of biological molecules, noninvasive diagnosis, and for tracking of real‐time processes in vivo and in vitro.^[^ ^128^ ^]^ To this end, AIE‐active materials offer certain advantages due to their high photoefficiency, antiphotobleaching properties, and the inherently better efficacy of solid‐state probes.^[^ ^96^ ^]^ AIE‐based fluorescent nanoparticles are more superior than other materials, particularly in terms of multifunctional targeting, low toxicity, high sensitivity, and better accuracy than the competing systems.^[^ ^129^ ^]^

#### 2.3.1 Biomolecules Detection

**Figure 8:**
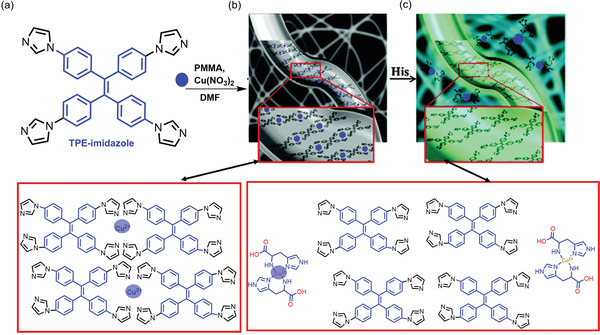
a) Scheme representing TPE‐imidazole Cu(II) complex‐doped nanofibrous membrane (IP‐Cu‐NMs) for the sensing of histidine (His). Molecular interactions in IP‐Cu‐NMs causing the b) luminescence off state, and c) luminescence on state when probing histidine (His). Adapted with permission.^[^ ^26^ ^]^ Copyright 2015, Royal Society of Chemistry.

In 2015, Kim et al.^[^ ^26^ ^]^ have leveraged the concept of AIE to synthesize TPE‐imidazole‐based Cu(II) electrospun nanofibrous membrane (termed IP‐Cu‐NMs) as a portable chemosensor for histidine (His) and His‐proteins, see **Figure** **8**a. The emission intensity of AIE‐active nanofibers was found to decrease when the Cu(II) concentration increases. Quenching of emission intensity is attributed to *π*−*π* stacking between the phenylene groups of TPE‐imidazole and Cu^2+^ cations, as depicted in Figure 8b. Upon adding histidine and His‐protein onto membrane surface, the emission intensity is enhanced significantly because histidine traps the Cu(II) and displaces it away from the TPE‐imidazole. The displacement of Cu(II) reduces *π*−*π* stacking between the phenylene groups of TPE‐imidazole and Cu(II), as shown in Figure 8c. This transformation switches on its luminescence through the interaction between two neighboring rings of TPE‐imidazole, hence restricting the rotational motion of phenylene rings.^[^ ^26^ ^]^

#### 2.3.2 Theranostic Studies

Porous materials can be employed as a drug delivery system for cancer treatment as they act as a “host” to quickly load targeted “guest” drug molecules, facilitated by their large surface area and tunable pore cavity.^[^ ^130^ ^]^ Recently, polymeric micelles are demonstrated as promising nanocarriers for drug delivery in cancer therapy. Polymeric micelles have several benefits over other nanoassemblies because of simple preparation, improved drug solubilization, biocompatibility, and biodistribution.^[^ ^131^ ^]^

**Figure 9:**
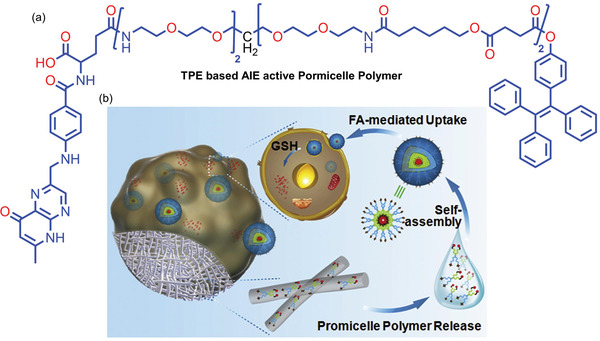
a) Structure of the TPE‐based promicelle polymer. b) Schematic representation of the implantable micelle‐generating depot for drug delivery. Folic acid (FA)‐mediated uptake is depicted prior to a controlled release when triggered by intracellular glutathione (GSH) level. Adapted with permission.^[^ ^24^ ^]^ Copyright 2018, Elsevier.

In 2017, Luo et al.^[^ ^24^ ^]^ developed an implantable micelle‐generating depot via blend electrospinning of AIE‐active promicelle polymers (**Figure** **9**a) into fibrous mats. The fibrous mats are subsequently implanted onto tumors through surgical or endoscopic means. Once fiber attaches to the interstitial medium, promicelle polymers are gradually released, as illustrated in Figure 9b. The whole process was visualized in vivo, thanks to the deployment of the AIE‐active promicelle polymers incorporating TPE derivatives (Figure 9a). Due to the self‐assembly of micelles, the phenyl rings in TPE come closer together, and their molecular motions become restricted, thereby increasing the radiative decay. This study shows the high promise of composite materials encompassing AIE‐active nanofibers for enabling mechanistic drug release to instigate theranostic innovations.

#### 2.3.3 Diagnostic Studies

Many deaths worldwide are linked to infections from bacteria and pathogens, which are causing severe medical and public concerns. As of now, methods such as polymerase chain reaction,^[^ ^132^ ^]^ immunoassays approach,^[^ ^133^ ^]^ and enzyme‐linked immunosorbent assay^[^ ^134^ ^]^ are robust, reliable, and user‐friendly techniques established in analytical laboratories, but these methodologies require costly apparatus and highly trained operators.^[^ ^135^, ^136^ ^]^ Electrospun nanofibrous mat provides a larger surface area, increasing the reaction rate and activation sites with potential for developing a compact and portable biosensor device.^[^ ^137^, ^138^ ^]^

**Figure 10:**
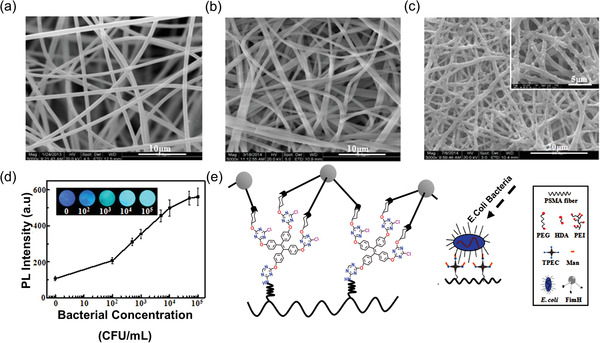
SEM images of a) PSMA and b) PSMA‐PEG‐TPEC‐Man; both fibers are randomly oriented and free of beads. c) PSMA‐PEG‐TPEC‐Man incubated with bacteria, showing a uniform distribution of bacteria over the fiber mat surface. d) Rising PL intensity of TPE after binding of *E. coli* bacteria at an increasing bacterial concentration. e) Biosensing mechanism proposed for bacterial detection. Reproduced with permission.^[^ ^25^ ^]^ Copyright 2015, American Chemical Society.

In 2015, Zhao et al.,^[^ ^25^ ^]^ by combining the photophysical properties of AIE materials, the binding capacity of mannose to *Escherichia coli* (*E. coli*) and the large surface area of nanofiber, designed electrospun fibrous mats based on the conjugates of TPE and mannose with sensitive turn‐on fluorescent sensing of *E. coli*. After fabrication of polystyrene‐*co*‐maleic anhydride (PSMA) fibers, they were immersed in PEG (polyethylene glycol) diamine solution at ambient temperature for 5 h to form the PSMA‐PEG fibers. The amine containing fibers were made to react with the solution of TPEC (tetraphenylethylene cyanuric chloride) in the presence of *N*,*N*‐diisopropylethylamine to generate the PSMA‐PEG‐TPEC fibers. Subsequently, these fibers were reacted with a solution containing mannose (Man) to yield the final probe, termed PSMA‐PEG‐TPEC‐Man. **Figure** **10**a,b shows that the PSMA and mannose‐conjugated PSMA fibrous mats were bead‐free, porous, and randomly oriented. The average fiber diameters were 0.86 ± 0.09 and 1.25 ± 0.11 µm, respectively. It was reported that the addition of spacers and mannose increases the fiber diameter, reducing the mat porosity from 91% to 81%. The scanning electron microscope (SEM) images of the fibers after incubation with bacteria display a relatively uniform distribution of microbes on the PSMA‐PEG‐TPEC‐Man (see Figure 10c).

Notably, Figure 10d shows that the fluorescence intensity of the PSMA‐PEG‐TPEC‐Man rises markedly with a higher concentration of *E. coli* bacteria. Here, it will be important to understand the underlying mechanism. In essence, the spacer between the PSMA electrospun mat and TPEC creates enough space to allow free rotation of phenyl rings in TPEC; therefore, the PL intensity is initially low. The covalently linked mannose binds to the *E. coli* bacteria, pulling the molecules closer together, which in turn increases the intermolecular interaction between the phenyl rings of TPEC (Figure 10e). Due to intermolecular interaction, the motion of phenyl rings becomes impeded, thus increasing PL intensity. Several studies with the same concept of grafting TPE derivatives with biomolecule‐sensitive luminophores (e.g., fluorescein and phloxine B) onto the electrospun nanofiber film for developing ratiometric, rapid, and susceptible film sensors, have immense potential for biomedical exploitations.^[^ ^139^, ^140^ ^]^

**Figure 11:**
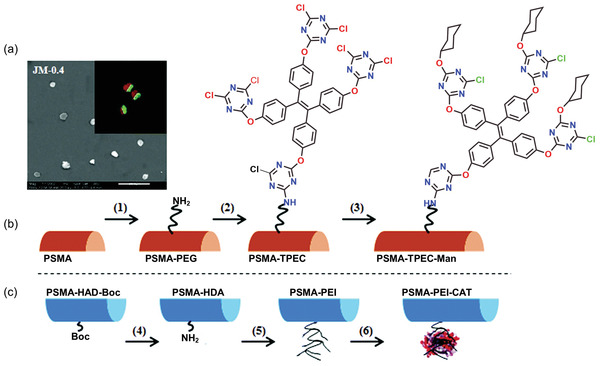
a) SEM image of Janus micromotors (JMs) with an aspect ratio of 0.4. Inset: CLSM image of JMs after labeling CAT with sulfo‐cyanine5 NHS (red) and labeling mannose with FITC (blue). b) Scheme representing a surface modification of Janus fiber rods with TPEC‐Man and CAT. c) Removal of Boc side group, followed by PEI grafting and CAT immobilization. (Abbreviations: PSMA—polystyrene‐co‐maleic anhydride; PEG—poly(ethylene glycol); PEI—poly(ethylene imine); catalase (CAT) is from bovine liver; TPEC—tetraphenylethylene cyanuric chloride; Man—mannose; HDA and Boc are the protecting groups). Adapted and reproduced with permission.^[^ ^141^ ^]^ Copyright 2019, Royal Society of Chemistry.

In 2019, the same group of Zhao et al.^[^ ^141^ ^]^ modified the fiber surface for low‐cost electrospun nanofibers based on Janus micromotors (JMs) and TPE derivative attached with mannose (TPEC‐Man) for sensitive detection of bacteria. Janus particles are considered next‐generation nanoparticles consisting of two distinct sides: hydrophilic and hydrophobic surfaces with a flexible structure.^[^ ^142^ ^]^ Janus fiber rods were fabricated by side‐by‐side electrospinning (Figure 3b) of modified polymers PSMA‐HDA‐Boc (HDA and Boc are protecting groups) and PSMA‐PEG in different compartments of fibers, followed by cryocutting into fiber rods with thicknesses of 2, 5, 10, 20 µm.^[^ ^143^, ^144^ ^]^ The surface of the Janus fiber rods was conjugated with TPEC, mannose (Man), and catalase (CAT) to form JMs. CLSM images confirmed the surface modification of the fiber rods after fluorescent labeling of CAT and mannose. The inset of **Figure** **11**a shows the compartmentalized distribution, indicating a well‐defined interface. Here, the PEG is used as a spacer between TPEC‐Man and polymer to increase conformational mobility, reducing the interaction between the JMs and bacteria (Figure 11b). Janus micromotors provide a “motion‐capture‐lighting” strategy by offering a fast and real‐time sensing of bacteria.^[^ ^141^ ^]^

The exemplars above demonstrate that the design and synthesis of bespoke AIE‐active system, when coupled with electrospinning, could be used to design and engineer high‐sensitivity bioprobes for specific biological targets.

### 2.4 Photodynamic and Photothermal Applications

The demand for freshwater is rapidly increasing with modernization evidenced worldwide. This presents a problem because the percentage of freshwater resources (2.5%) is scarce compared to seawater resources (97.5%).^[^ ^145^, ^146^ ^]^ To this end, the conversion of seawater to freshwater by desalination is a possible route for generating freshwater supply to meet the world's ever‐increasing demand.^[^ ^147^ ^]^ Solar vapor evaporation has been proposed for clean water generation as solar energy is one of the most ecofriendly renewables, and this technique can be deployed in off‐grid areas. However, solar desalination requires a good solar steam efficiency.^[^ ^148^, ^149^ ^]^ This requirement might be met by nanofibers, which provide a large surface area and porous network for boosting the solar steam efficiency^[^ ^150^, ^151^ ^]^ compared with other competing materials, such as carbon foams, anodized aluminum oxide substrates, natural wood, and hydrogels.^[^ ^150^, ^152^, ^153^ ^]^ The electrospun nanofibers may result in a denser film or membrane mat with a small pore size that hinders water transportation.^[^ ^154^, ^155^ ^]^ Instead, the low density and high porosity of an aerogel composite may offer a suitable solution,^[^ ^156^ ^]^ combined with AIEgens with a rotor‐like structure acting as an efficient photothermal agent.^[^ ^157^ ^]^

**Figure 12:**
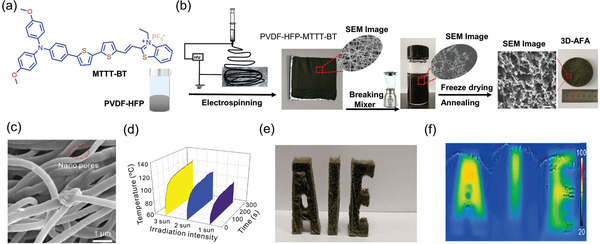
a) Left: Chemical structure of MTTT‐BT. b) Processing steps involved in fabrication of 3D all fiber aerogel for solar steam generation. Electrospinning was conducted with a flow rate of 0.8 mL h^−1^ at 15 kV. c) SEM image of 3D AFA. d) Temperature histories show the temperature rise of 3D AFA when exposed to 1, 2, and 3 suns. e) 3D AFA molded into AIE letters, and f) photothermal imaging under sunlight irradiation. Adapted with permission.^[^ ^158^ ^]^ Copyright 2020, American Chemical Society.

In 2020, Li et al.^[^ ^158^ ^]^ demonstrated an approach to combine AIE properties of MTTT‐BT ((E)‐2‐(2‐(5″‐(4‐(bis(4‐methoxyphenyl)amino)phenyl)‐[2,2″‐bithiophen]‐5‐yl)vinyl)‐3‐ethylbenzo[d]thiazol‐3‐ium, **Figure** **12**a), with the large specific area of nanofiber and high porosity of aerogels to fabricate an “all‐fiber aerogel” (AFA) for solar vapor evaporation of seawater. Figure 12b summarizes the material processing steps involved. PVDF‐HFP (poly(vinylidene fluoride‐*co*‐hexafluoropropylene)) (18 wt%) was first prepared in a solution mixture of THF:DMF (v/v = 3.7), before introducing the powders of MTTT‐BT. Figure 12c shows a 2D planar architecture of the electrospun nanofiber mat, which has no bead formation. Subsequently, the 2D structure was reconstructed to a 3D all fiber aerogel (3D AFA) achieving an exceedingly low density of 0.01 g cm^−3^. The aerogel was fabricated by blending electrospun nanofiber with a water and *tert*‐butanol mixture to form a uniform dispersion of short nanofibers. The dispersion was then poured into a mold and frozen in liquid nitrogen for 30 min, before freeze‐drying for 48 h to form 3D AFA. To create a bifunctional layer, the 3D AFA was exposed to oxygen plasma, electrosprayed by a gelatin solution, then crosslinked to glutaraldehyde to form a permanently hydrophilic surface. The efficacy of photothermal conversion employing 3D AFA had been tested under 1, 2, and 3 sun irradiance levels. Under 2 and 3 suns, as shown in Figure 12d, the maximum temperatures reached were about 107 and 120 °C, respectively. The highly porous 3D AFA has a low thermal conductivity, it can be molded into bulk shapes (Figure 12e) with photothermal capacity under sunlight irradiation (Figure 12f).

**Figure 13:**
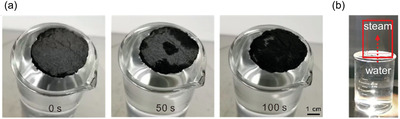
a) Time‐lapse photographs of 3D AFA floating on the water surface show water pumping through the hydrophobic layer and wetting the top surface by hydrophilic interaction. b) Steam generation (highlighted in red box) by solar irradiation. Adapted with permission.^[^ ^158^ ^]^ Copyright 2020, American Chemical Society.

**Figure** **13**a shows how the bifunctional surface of 3D AFA permits water transportation from the bottom hydrophobic layer across the upper hydrophilic layer, while the hydrophobic layer enables the 3D AFA to float on water. The upper hydrophilic layer is beneficial for “pumping” water through the interconnected porous network within the aerogel, resulting in a continuous flow of water without direct contact with the bulk water found underneath. Here, the hierarchically porous structure with interconnected nanopores facilitates the water transportation and vaporization within the microchannels. Interestingly, the results reveal that AIEgens with rotors and loose packing, where molecular motion is possible even in the aggregated state can act as an efficient photothermal agent.^[^ ^158^ ^]^ This effect is shown in Figure 13b, where solar irradiation of 3D AFA affords the conversion of solar energy to heat for steam generation.

**Figure 14:**
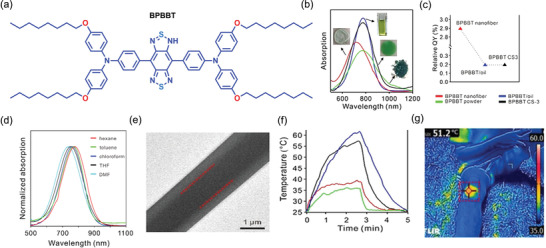
a) Chemical structure of BPBBT. b) Absorption spectra of BPBBT and its composites (green line—BPBBT powder, red line—BPBBT fiber, blue line—BPBBT‐oil solution, and black line—BPBBT CS‐3). c) Quantum yields of BPBBT in oil, BPPBT nanofiber, and CS‐3 fibers. d) The blue‐shifted absorption peak of BPBBT when increasing the polarity of the solvent. e) TEM images of CS‐3. f) Change in temperature as a function of irradiation time by one sun power. g) Infrared image of the CS‐3 photothermal patch exposed to natural sunlight for 1 min. (Abbreviations: BPBBT ‐ 4,4'‐(1H‐2λ^3^‐benzo[1,2‐c:4,5‐c']bis([1,2,5]thiadiazole)‐4,8‐diyl)bis(N,N‐bis(4‐(octyloxy)phenyl)aniline)). Adapted with permission.^[^ ^31^ ^]^ Copyright 2020, Wiley‐V.

Also in 2020, Li et al.,^[^ ^31^ ^]^ by reversing the principle of AIE, showed an intriguing pathway for improving the molecular motions of AIE‐active molecules to boost the photothermal efficiency of electrospun fibers. The coaxial electrospinning technique (Figure 3d) can be harnessed to fabricate core–shell electrospun nanofibers. AIE‐active molecule containing a twisted structure with more rotors can serve as an excellent photothermal agent. Fabrication of the core–shell fiber with AIE‐active material containing oil as core phase and polymer as shell, retains the molecular motion of the materials. Due to the molecular motion of the rotor in the AIE‐active materials, the nonradiative decay increases, which significantly enhances photothermal efficiency. For the fabrication of the core–shell fiber (named CS‐3), the AIE‐active molecule (BPBBT, see **Figure** **14**a) was dissolved in olive oil as a core phase, and pure PVDF‐HFP (18 wt%) solution in DMF/THF mixture (7:3, v/v) as a shell phase. The core–shell fiber has a broad absorption band in the range of 600−900 nm, with a maximum at 780 nm that is useful for harvesting the sun rays and turning them into usable energy. Given that BPBBT exhibits negative solvatochromism, the significant polarity of PVDF‐HFP may be the reason of the blue‐shift evidenced in the absorption spectra of BPBBT nanofiber (Figure 14b). Negative solvatochromism is supported by the blue‐shifting of BPBBT's absorption band with an increasing solvent polarity (Figure 14d). The quantum yield of CS‐3 and BPBBT in oil is less than the core–shell fibers of BPBBT in PVDF‐HFP, indicating the lower radiation decay due to an increase in the molecular motion of BPBBT in oil (Figure 14c). Transmission electron microscope (TEM) images clearly show the continuous and uniform fibers with the core–shell structure (Figure 14e). On irradiation of BPBBT fiber, BPBBT powder, BPBBT in oil, and CS‐3 with a power density of 1 kW m^−2^ ( = 1 sun of irradiation), the temperature of the CS‐3 core–shell fiber mat and BPBBT in oil was determined to increase to 61.1 and 56 °C, respectively (Figure 14f). The increase of the temperature of core–shell fiber reveals that BPBBT in oil plays a significant role in boosting the photothermal efficiency of the resultant fibers. The core–shell fibrous patch was applied to a volunteer's knee and exposed to natural sunlight for 1 min. The temperature of the patch increased to 51 °C (Figure 14g), which is much higher than the normal skin and body temperature. This demo demonstrates the promising application of such a photothermal patch for controlled drug release and therapeutic studies.

**Figure 15:**
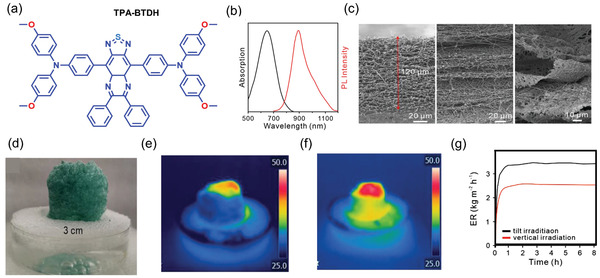
a) Chemical structure of TPA‐BTDH. b) PL and absorption spectra of TPA‐BTDH in THF (tetrahydrofuran) solution. c) SEM images showing the cross‐section morphologies of TPA‐BTDH/PMMA nanofibrous mat before (left) and after the treatment with 1 m NaBH_4_ solution for 10 min (middle) and after 60 min (right). d) Photograph of AFPCF wrapped in foam and floating on water. Infrared imaging revealing the temperature rise subject to the e) vertical and f) tilt irradiation, employing 1 sun power. g) Evaporation rate (ER) of an AFPCF sample tested for 8 h under tilt and vertical irradiation with 1 sun power. (Abbreviations: TPA‐BTDH ‐ 4,4'‐(6,7‐diphenyl‐4a,8a‐dihydro‐1H‐2λ^3^‐[1,2,5]thiadiazolo[3,4‐g]quinoxaline‐4,9‐diyl)bis(N,N‐bis(4‐methoxyphenyl)aniline); PMMA ‐ poly(methyl methacrylate; AFPCF ‐ all‐fiber porous cylinder‐like foam). Adapted with permission.^[^ ^159^ ^]^ Copyright 2021, Wiley‐VC.

Furthering the innovative concept of AIE reversal, in 2021, Tang and co‐workers^[^ ^159^ ^]^ fabricated an all‐fiber porous cylinder‐like foam (AFPCF). The AFPCF is a composite material that incorporates the AIE‐active molecule called TPA‐BTDH, as shown in **Figure** **15**a. The strong and broad absorption band (max at 645 nm) of TPA‐BTDH is beneficial for absorbing sunlight energy, and its maximum PL emission is at 908 nm in the near‐infrared (NIR) region (Figure 15b). This AIE‐based photothermal agent has the capacity for ROS generation to simultaneously accomplish solar steam generation and antibiofouling. Recently the use of the simulated tilt irradiation technique has gained attention, compared with the conventional simulated sun positioned vertically above the surface of the evaporator during evaporation test. Simulated tilt irradiation allows the sample's top and side surfaces to absorb solar energy, thus better emulating the exposure by the natural sunlight.^[^ ^160^, ^161^, ^162^ ^]^ AFPCF comprises an interconnected porous hydrophilic network of PMMA polymer for transporting pumped water across the surface, and a side area‐assisted evaporation system for enhancing evaporation and effective antibiofouling function. The twisted nonplanar structure and molecular rotor groups within the fibers favor molecular motion, resulting in improved photothermal conversion and higher ROS generation under sunlight irradiation.

The hydrophilicity of the PMMA fiber was increased by treatment with plasma and expanded in NaBH_4_ solution (1 m) for different time intervals. From the SEM characterization shown in (Figure 15c), it can be seen that the gap between the layers of AFPCF increases as the thickness of the layer decreases, scaling with the expansion time. To test its solar steam generation capacity, a 3 cm thick AFPCF was wrapped in foam and floated in a water solution containing 3.5 wt% NaCl (Figure 15d). As shown in Figure 15e, when the evaporator setup was irradiated vertically with 1‐sun power for an hour; the surface temperature increased to 46.5 °C but its side temperature was substantially lower at ≈29 °C. In contrast, when the test was conducted under tilted irradiation for the same duration, both the evaporator's top and side temperatures increased to 44.5 and 39.5 °C, respectively, see Figure 15f. Significantly, when the nanofiber was exposed to tilted irradiation, the evaporation rate had increased to 3.6 kg m^−2^ from 2.4 kg m^−2^ for vertical irradiation (Figure 15g). The results suggest that the side area‐assisted irradiation boosts overall evaporation. AFPCF also generates ROS upon exposure to sunlight, enabling it to act as an antibiofouling evaporator system.^[^ ^159^ ^]^

**Figure 16:**
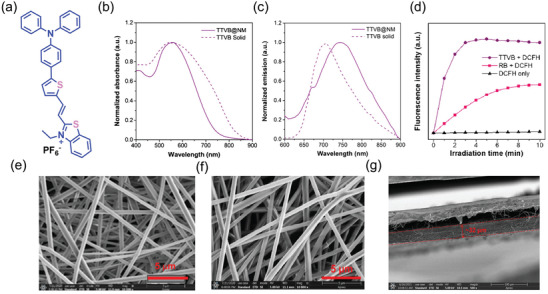
a) Chemical structure of TTVB. b,c) Absorption and emission spectra of TTVB@NM and TTVB solids. d) Comparing the ability of TTVB and a commercially available photosensitizer (RB, rose Bengal) to generate ROS in an aqueous solution under light irradiation (34 mW cm^−2^) with the DCFH indicator. SEM images of e) nanofibrous membrane (NM), f) TTVB@NM, and g) cross‐sectional profile showing the multilayered nanofibrous membrane of TTVB@NM with a thickness of ≈30 µm. Adapted with permission.^[^ ^30^ ^]^ Copyright 2021, Elsevier.

AIE‐active materials are good photosensitizers because of their ability to generate singlet oxygen in the aggregated state, combined with strong fluorescence efficiency, large Stokes shift, good biocompatibility, and resistance to photobleaching.^[^ ^163^, ^164^ ^]^ These properties make AIE materials promising for bacterial killing, cancer treatment, and imaging. Twisted AIE materials with donor and acceptor (D−A) moiety have been demonstrated as high ROS generator materials due to their efficient ISC capacity.^[^ ^165^, ^166^ ^]^ Recently in 2021, Li et al.^[^ ^30^ ^]^ reported the electrospun nanofibrous membrane (NM) termed TTVB@NM, comprising PVDF‐HFP polymer doped with the AIEgen, called TTVB ((E)‐2‐(2‐(5‐(4‐(diphenylamino)phenyl)thiophen‐2‐yl)vinyl)‐3‐ethylbenzo[d]thiazol‐3‐ium, **Figure** **16**a). The solid material of TTVB has a broad absorbance range (400–700 nm), with an absorption maximum of 552 nm, which will be advantageous for sunlight‐triggered ROS generation; it exhibits a fluorescence maximum of 700 nm (Figure 16b,c). The ROS production efficiency of TTVB was investigated using dichlorofluorescein (DCFH) indicators when exposed to a white light source comparable to natural sunlight. In terms of ROS generation under simulated solar irradiation, the performance of TTVB surpasses that of Rose Bengal (RB, a commercial photosensitizer) (Figure 16d). The nanofiber TTVB@NM was demonstrated to be effective as an antimicrobial film subject to sunlight irradiation. TTVB has the D−*π*−A type structure, consisting of a TPA group acting as electron donor, vinylthiophene group as the *π*‐spacer group, and 3‐ethylbenzo[*d*]thiazol‐3‐ium as the electron acceptor. From the infrared spectra of TTVB@NM, a new band was detected around 1585 cm^−1^ corresponding to the C=C stretching mode, indicative of the doping of TTVB within nanofibers.

**Figure 17:**
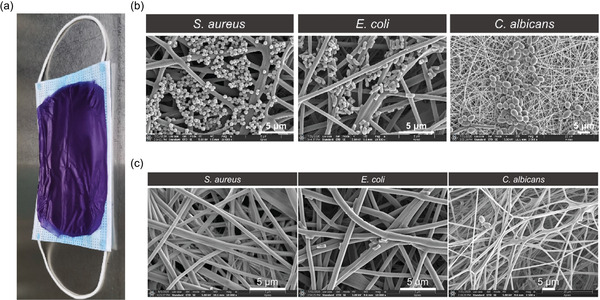
a) A medical facemask coated with TTVB@NM. b) FESEM images of three different microbes adhering on the surface of the TTVB@NM, and c) their elimination from fiber surface after simulated sunlight irradiation. Adapted with permission.^[^ ^30^ ^]^ Copyright 2021, Elsevier.

TTVB@NM has multilayered nanofibrous morphology with no other significant microstructural changes compared with the pristine NM (Figure 16e,f). The average fiber diameter of TTVB@NM was found to be ≈550 nm, which is relatively larger than that of the NM (≈480 nm). The thickness of the membrane is ≈30 µm with an electrospinning time of 30 min (Figure 16g). TTVB@NM exhibits an 80% inhibition efficacy under simulated sunlight. TTVB@NM was applied onto a disposable facemask for practical application test (**Figure** **17**a), and sprayed with aerosols of three different microbes—*E. coli*, *S. aures*, and *C. albicans*, for 10 s at an air flow rate of 0.2 mL min^−1^. Using the field emission SEM (FESEM), the pathogens can be clearly observed on the fibers, see Figure 17b. However, after irradiation with simulated sunlight (10 min at 34 mW cm^−2^) due to the antimicrobial activity of TTVB@NM, only a few pathogens survived on the surface of the fibrous membrane, see Figure 17c. Even under natural sunlight, TTVB@NM requires only 5−10 min for effective killing of pathogens.^[^ ^30^ ^]^

In 2022, Dong et al.^[^ ^167^ ^]^ created an antibacterial, biocompatible AIE nanofibrous patch that eliminates multidrug‐resistant bacterial infection and enhances wound healing. The AIE‐active nanofiber demonstrates effective antibacterial action against *Staphylococcus aureus* and methicillin‐resistant *Staphylococcus aureus* due to the AIG‐ROS characteristic of AIEgens. The results so far suggest that AIE nanofibrous dressing technology may yield a simple, low‐cost, and efficient method for clinical implementations such as personalized therapies.

### 2.5 AIE Electrospun Nanofiber Composites for Multistimuli Sensing

Stimuli‐responsive solid‐state luminescent materials have gained attention in recent years due to their widespread applications, encompassing biochemical sensors, environmental conditions sensors (temperature, pressure, pH, and viscosity), and volatile organic compound (VOC) sensors, toxic gases sensors, and wearable chemosensors. Due to their inherent property, AIEgens are effectively utilized for making luminescent films sensors.^[^ ^38^, ^168^, ^169^ ^]^ The twisted donor and acceptor type of AIE materials exhibit distinct types of excitation transition states (Figure 4), specifically the LE state, intramolecular charge transfer state (ICT), and HLCT state. The HLCT state has both the LE and ICT components.^[^ ^170^, ^171^ ^]^

#### 2.5.1 Vapor Sensing

**Figure 18:**
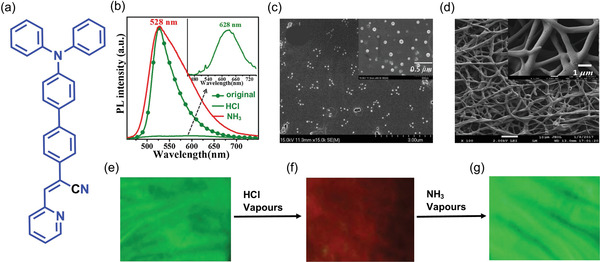
a) Chemical structure of DBPA. b) PL spectra of DBPA upon exposure to the vapors of HCl and NH_3_, respectively (Inset: enlarged part of DBPA fumed with HCl vapor). SEM images of c) nonporous film (drop‐casting technique) and d) porous film (electrospinning technique). Fluorescent images of electrospun nanofiber of DBPA@SEBS under UV 365 nm for the e) pristine state, f) after exposure to HCl vapor, and g) recovery of green emission when exposed to NH_3_ vapor. (Abbreviations: DBPA ‐ (Z)‐2‐(4'‐(diphenylamino)‐[1,1'‐biphenyl]‐4‐yl)‐3‐(pyridin‐2‐yl)acrylonitrile); HCl ‐ hydrochloric acid; SEBS ‐ poly[styrene‐b‐(ethylene‐co‐butylene)‐b‐styrene]). Adapted with permission.^[^ ^172^ ^]^ Copyright 2018, Elsevier.

In 2017, Zhang et al.^[^ ^172^ ^]^ synthesized a TPA‐derivative DBPA (**Figure** **18**a), in which pyridine acts as a recognition site to achieve the HLCT transition state. High PLQY was reported, up to ≈76% and ≈83% for its crystalline state and electrospun nanofiber membranes, respectively. Due to the larger steric hindrance, DBPA is protonated on the pyridine nitrogen (rather than the TPA nitrogen) in the presence of HCl. After 30 s of exposure to HCl vapors (80 ppm), the powder samples of DBPA under ambient light change color from yellow to dark red, corresponding to a 100 nm red‐shift, while its green emission is quenched. When exposed to NH_3_ vapor, the green emission with the broad tail can be reversed (Figure 18b).

The thin film was fabricated by drop‐casting (Figure 18c) and electrospinning methods using SEBS (poly[styrene‐*b*‐(ethylene‐*co*‐butylene)‐*b*‐styrene]) triblock copolymer to yield a flexible material for practical application. The SEBS polymer was chosen because this is an elastomer with good aging resistance and high elasticity. From FESEM analysis, it was observed that the nanofibers exhibit a highly porous randomly oriented mesh architecture with an average diameter of 650 ± 100 nm (Figure 18d). In the presence of HCl vapor, the emission color switches from green to red, and on ammonia exposure, it reverts to green (Figure 18e–g). On the detection limit, the results indicate that the larger surface area of the porous electrospun nanofibers has dramatically enhanced the HCl sensing capability of the probe compared to the nonporous film obtained by drop casting.^[^ ^172^ ^]^ Also in 2017, Cheng et al.^[^ ^173^ ^]^ created a humidity sensor using the TICT effect of AIEgen. The AIEgens are microenvironmentally sensitive, whereas the polymer in the fiber acts as a network for capturing moisture.

**Figure 19:**
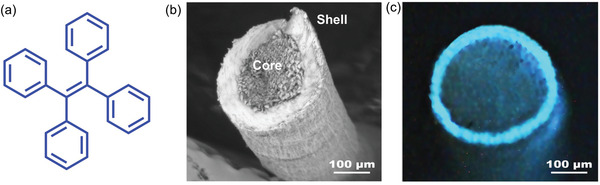
a) Chemical structure of TPE. b) SEM images of the cross‐section of the AIE‐active fiber, showing the core–shell morphology. c) Photoluminescence of the fiber under UV 365 nm. Adapted with permission.^[^ ^174^ ^]^ Copyright 2021, Elsevier.

In 2021, Jiang et al.^[^ ^174^ ^]^ fabricated a core–shell type electrospun nanofiber based on TPE as the AIEgen (**Figure** **19**a) with potential use in display, anticounterfeiting, and hazardous gas detection. The TPE nanofiber was fabricated by the conjugate electrospinning method, employing thermoplastic PU and polyethylene terephthalate (PET) polymers; the latter polymer provides mechanical flexibility.

The conjugate electrostatic spinning method depends on electrospinning parameters such as spinning solution concentration, spinning rate, traction rate, and guest concentration. AIE‐active fibers and luminescent textiles with excellent flexibility can be fabricated by optimizing the electrospinning parameters. Fluorescence intensity was found to be directly proportional to the coverage of the fiber under optimal spinning conditions. SEM characterization reveals that the fluorescent nanofiber has a core–shell structure (Figure 19b), and under the stereoscopic microscope with a 365 nm excitation source it emits an intense blue light (Figure 19c).

**Figure 20:**
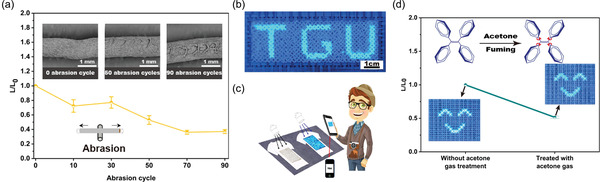
a) Fluorescence intensity ratio of AIE‐active fiber before and after abrasion, and morphology of fiber without abrasion, and after 50 and 90 abrasion cycles. b) PL image of AIE‐active fiber woven on cloth as the letters were excited under 365 nm UV lamp. c) Schematic representation of AIE‐active fiber applied in anticounterfeiting technology. d) Change in PL intensity of luminescent textile before and after exposure to acetone vapor for 10 min. Reproduced with permission.^[^ ^174^ ^]^ Copyright 2021, Elsevier.

For practical use in textile industries, the washability and durability of fibers are important properties. **Figure** **20**a shows that after 90 abrasion cycles, the fluorescence intensity of the core–shell fiber has fallen to 40%, because the surface fluorescent layer of nanofiber has been worn off or heavily deformed. The study further demonstrates interesting demos (Figure 20b,c), whereby the AIE‐active fibers are woven on the cloth and utilized as a QR (quick response) code or barcode for electronic devices in security applications. Figure 20d shows the potential of using the luminescent textile for sensing acetone vapors. In the presence of acetone, the phenyl rings of TPE rotate freely, thereby increasing the nonradiative decay but decreasing the fluorescence emission intensity by ≈50%.^[^ ^174^ ^]^

#### 2.5.2 Pressure and Mechanical Stress Sensing

Pressure‐sensing materials are required for emergent smart applications, such as structural health monitoring, direct visualization of stress intensity in wind tunnel tests, and nondestructive evaluation in aircraft industries.^[^ ^172^, ^175^, ^176^ ^]^ Such pressure‐sensing applications could exploit the unique properties of ML materials,^[^ ^27^ ^]^ for their emission characteristics alter or respond when subject to a mechanically induced force, stress, or pressure. Because of their increased fluorescence intensity in the solid or aggregated form, AIE‐based ML materials are favorable to yield a turn‐on type pressure probe. Furthermore, because of their twisted geometry and the existence of space in the crystal lattice, they are intrinsically sensitive to stress (defined as force per unit area, with units of Pascal (Pa)).^[^ ^28^ ^]^

**Figure 21:**
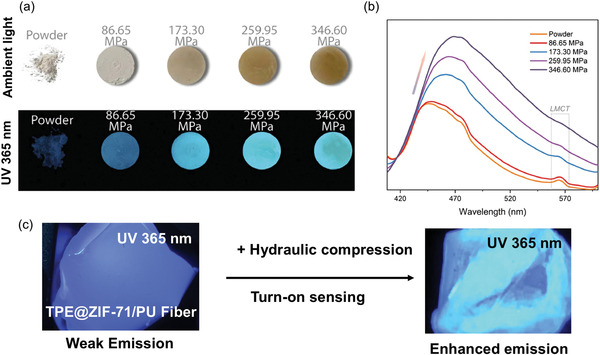
a) TPE@ZIF‐71 pellets prepared under different pressures, their colors viewed under ambient light and under a 365 nm UV lamp. b) Emission intensity rises with mechanical stress level indicative of mechanofluorochromic turn‐on sensing behavior. c) Turn‐on mechanofluorochromism of TPE@ZIF‐71/PU fibers subject to a uniaxial compression generated by hydraulic press. Adapted and reproduced under the terms of the CC‐BY creative Common Attribution 4.0 International License.^[^ ^182^ ^]^ Copyright 2022, Elsevier.

TPE has a highly twisted conformation held together by many CH−*π* and CH−C short intermolecular interactions. These short‐range interactions are mechanically sensitive to a force stimulus with the propensity for altering spectroscopic properties, particularly photoluminescence spectra.^[^ ^177^ ^]^ Yuan et al.^[^ ^178^ ^]^ in 2014 investigated the mechanochromic behavior of TPE crystals as a function of pressure, and found that a low pressure of up to 1.5 GPa reduces TPE emission due to an increase in the nonradiative decay. From 1.5 to 5.3 GPa, the emission intensity of TPE increases with pressure. In the 1.5–5.3 GPa pressure range, molecules are close enough to form and strengthen hydrogen bonds between the aromatic rings, restricting *π*−*π* interactions.^[^ ^179^ ^]^ Many researchers have attempted to synthesize TPE derivatives to improve and optimize their mechanofluorochromic characteristics.^[^ ^180^, ^181^ ^]^ However, these approaches had a limited success, and the complexity of the proposed synthesis routes further hindered their progress. The confinement of TPE in a nanoporous framework structure can modulate the short intermolecular contacts, improving the mechanoluminescent character of TPE. In 2022, Zhang et al.^[^ ^182^ ^]^ demonstrated the nanoconfinement of TPE in zeolitic imidazolate framework‐71 (ZIF‐71 is a metal‐organic framework (MOF) material), where the encapsulation of the TPE guest molecules by the ZIF‐71 host was achieved by a bottom‐up synthetic approach. TPE@ZIF‐71 shows a “turn‐on” type mechanofluorochromic sensing response (i.e., PL rises with stress) and the sensitivity of TPE has been amplified by a factor of tenfold. **Figure** **21**a shows the emission intensity of TPE@ZIF‐71 pellets increases with elevating pressure.

Remarkably, the nanoconfined TPE exhibits a turn‐on type mechanofluorochromic response even under a relatively low nominal stress of under 350 MPa, thereby overcoming the ACQ effect evidenced in isolated TPE crystals observed earlier by Yuan et al.^[^ ^178^ ^]^ Turning to TPE@ZIF‐71, as pressure increases the TPE molecules within the ZIF‐71 pore become further constrained, giving a more severe caging effect. Synchrotron far‐infrared (terahertz) spectroscopy and X‐ray diffraction (XRD) revealed that the applied pressure causes framework deformation and structural amorphization of ZIF‐71. It was reasoned that the mechanical collapse of the host framework enables TPE guests to form aggregates, thereby enhancing the emission of TPE with applied stress (Figure 21b). Furthermore, TPE@ZIF‐71 was combined with PU for electrospinning to fabricate the TPE@ZIF‐71/PU fibers. Figure 21c shows that the resultant fibrous membrane can potentially be employed as a turn‐on mechanofluorochromic 2D sensor. This study suggests that nanoconfinement of AIEgens in MOFs, to yield the “AIE@MOF” type systems, will confer unconventional photophysical and photochemical properties.^[^ ^14^ ^]^ In contrast, several previous studies have considered the use of AIE molecules acting as a bridging linker in the synthesis of crystalline MOF structures.^[^ ^183^, ^184^ ^]^

**Figure 22:**
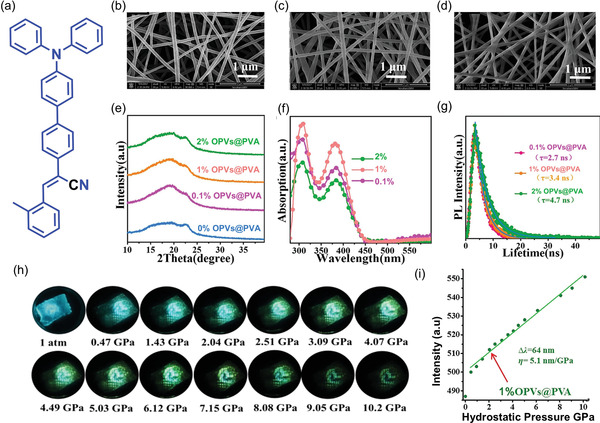
a) Chemical structure of AN. b–d) SEM images of electrospun fibers of AN@PVA with different dye loadings (0.1, 1, and 2 wt%), respectively. e) Comparing the XRD patterns of the bare (0 wt%) PVA nanofiber with dye‐doped nanofibers. f) Absorption spectra of the nanofibers. g) Lifetime decay of the luminescent nanofibers. h) PL photographs of AN@PVA nanofibrous film (1 wt%) under compression at different pressure levels (ambient to 10.2 GPa) and excited under a 365 nm UV lamp. i) Plot representing absolute intensity of maximum fluorescence band versus hydrostatic pressure of 1 wt% of AN@PVA; a best‐fitted line to points with *R* ^2^ = 0.9894 for Δ*λ* = *λ*−*λ*_0_, in which Δ*λ* is the color shift determined from PL spectra at pressures of 10 GPa and 1 atm, respectively. (Abbreviations: AN ‐ (Z)‐2‐(4'‐(diphenylamino)‐[1,1'‐biphenyl]‐4‐yl)‐3‐(o‐tolyl)acrylonitrile; PVA ‐poly(vinyl alcohol)). Adapted with permission.^[^ ^27^ ^]^ Copyright 2018, Wiley‐VCH.

In 2018, Yang et al.^[^ ^27^ ^]^ reported a ratiometric piezochromic electrospun nanofiber composite, designated as AN@PVA. It comprises AIE‐active donor–acceptor type cyano‐substituted oligo(*p*‐phenylenevinylene) dye molecules AN (**Figure** **22**a), integrated into poly(vinyl alcohol) (PVA) polymer with different mass concentrations (0.1, 1, and 2 wt%). SEM micrographs in Figure 22b–d show that the width of the individual fiber is ≈150 nm, with a smooth surface morphology and without beads formation. The XRD pattern of the bare PVA film has two broadened diffraction peaks unchanged in AN@PVA films, indicating that AN was dispersed into PVA in amorphous form (Figure 22e). Absorption spectroscopy of the electrospun films (Figure 22f) did not reveal any notable spectral changes compared to the bare AN, indicating no change in the molecular conjugation length. However, the increase in the PL decay lifetime of AN@PVA as a function of dye loading (see Figure 22g) indicates the rise in intermolecular interactions between the neighboring molecules. Under mechanical compression, it can be seen in Figure 22h that the PL emission of the 1 wt% AN@PVA nanofibrous film exhibited a bathochromic shift, switching from light blue to green, due to the enhanced exciton coupling of adjacent molecules. By tracking the emission maxima at different hydrostatic pressures, the mechanoluminescent detection sensitivity of 1 wt% AN@PVA was found to be 5.1 nm GPa^−1^. Figure 22i shows a linear correlation between emission intensity as a function of pressure. The combined effect of intermolecular interaction under compression with the planarization of phenyl rings may play a role in the observed piezochromic behavior of the molecules.^[^ ^27^ ^]^ The findings are fascinating but the exact mechanism is yet to be fully understood. The fiber provide the

#### 2.5.3 Explosive Sensing

**Figure 23:**
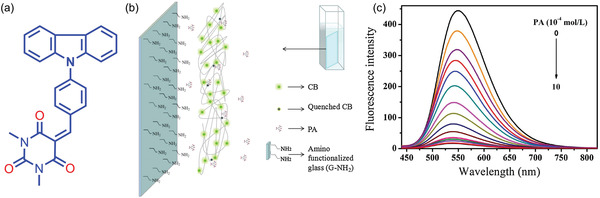
a) Chemical structure of CB. b) Schematic representation of CB‐PS/G‐NH_2_ picric acid sensor. c) Emission spectra of CB‐PS/G‐NH_2_ in the presence of an increasing concentration of PA (0 ×  10^−4^ to 10  ×  10^−4^ m) in water. (Abbreviations: CB ‐ 5‐(4‐(9H‐carbazol‐9‐yl)benzylidene)‐1,3‐dimethylpyrimidine‐2,4,6(1H,3H,5H)‐trione; PS ‐ polystyrene) Adapted with permission.^[^ ^185^ ^]^ Copyright 2018, Elsevier.

In 2018, Cui et al.^[^ ^185^ ^]^ fabricated electrospun nanofiber membrane (CB‐PS) (**Figure** **23**a) on amino‐functionalized glass (G‐NH_2_) for nitroaromatic explosive sensing. The CB‐PS nanofibers were electrospun from a solution of DMF:THF (3:1 v/v), comprising the AIE‐active carbazole derivate dispersed in polystyrene (PS) polymer. Figure 23b depicts the resultant sensor being used for the selective and sensitive detection of picric acid in an aqueous solution, where its fluorescence quenching behavior was studied. The PL spectra of CB‐PS/G‐NH_2_ with an increasing concentration of picric acid in water were obtained to investigate the sensing ability of the CB‐PS/G‐NH_2_. Figure 23c shows that the emission intensity gradually decreases (i.e., turn‐off type luminescent quenching) as the concentration of picric acid increases.

#### 2.5.4 Molecular Recognition

**Figure 24:**
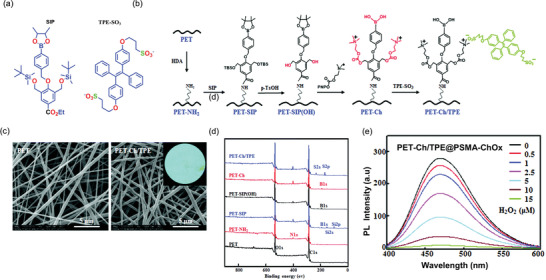
a) Scheme for PET‐Ch/TPE fiber. b) Chemical structures of SIP and TPE‐SO_3_. c) SEM images of electrospun fibers of PET and PET‐Ch/TPE, showing a similar network morphology. d) XPS spectra of all the fibers (PET, PET‐NH_2_, PET‐SIP, PET‐SIP(OH), PET‐Ch, and PET‐Ch/TPE. e) Fluorescence spectra of PET‐Ch/TPE@PSMA‐ChOx fibers after incubation with H_2_O_2_ at different concentrations for 12 min. Adapted with permission.^[^ ^188^ ^]^ Copyright 2019, Royal Society of Chemistry.

Visual probes for sensing biomolecules and active substances exploiting AIE‐active materials are increasingly studied due to their high photostability and quantum yield, low photobleaching, and facile tunability.^[^ ^186^, ^187^ ^]^ In 2019, Zhao et al.^[^ ^188^ ^]^ designed and fabricated a self‐propagating luminescent probe (**Figure** **24**a) for visualizing hydrogen peroxide (H_2_O_2_) and choline (Ch). The electrospun fibrous mats are made from either PET (polyethylene terephtalate) or PSMA (polystyrene‐co‐maleic anhydride) polymers with TPE molecules. The PET‐Ch/TPE fibers were fabricated sequentially grafting of self‐immolative probes (SIP) on modified aminated PET fibers (PET‐NH_2_) to form PET‐SIP. Choline is conjugated on PET‐SIP fiber via an acylation reaction to form PET‐Ch, then TPE‐SO_3_ was attached on PET‐Ch through electrostatic complexation to yield PET‐Ch/TPE fibers. On the other hand, the PSMA‐ChOx (choline oxidase) fibers were fabricated by modifying PSMA fibers with PEI linkers to conjugate ChOx, as depicted in Figure 24b.

While the surface morphology of the modified fiber (PET‐Ch/TPE) is reminiscent of the pristine PET (Figure 24c), the size of the former (0.48 ± 0.08 µm) is marginally higher than the latter fiber (0.36 ± 0.08 µm). The changes to the fiber surface were examined by X‐ray photoelectron spectroscopy (XPS), the results are shown in Figure 24d. From XPS, it was found that PET‐NH_2_ fiber showed a new weak peak of N 1s at the binding energy of ≈400 eV, and when SIP was grafted on PET‐NH_2_ fiber, three new peaks of B 1s, Si 2s, and Si 2p appeared. Two peaks disappeared when choline was grafted on the PET‐SIP fiber. After TPE‐SO_3_ was attached to the fiber, two new peaks of S 2s and S 2p were detected in PET‐Ch/TPE fibers. Finally, the PET‐Ch/TPE and PSMA‐ChOx fibers were assembled to form the “PET‐Ch/TPE@PSMA‐ChOx” composite mat for the sensitive detection study of H_2_O_2_. PET‐Ch/TPE fibers exhibited fast fluorescence fading in 12 min after assembly with PSMA‐ChOx fibers as the H_2_O_2_ concentration rose from 0 to 15 m (Figure 24e). Notably, the sensitivity to detect H_2_O_2_ is enhanced in the assembled form. The detection limit of the composite fibrous mat was 0.5 × 10^−6^ m H_2_O_2_ with a fluorescence fading time of 30 min due to the self‐propagating reactions of PSMA‐ChOx. The composite mat could also sense choline at low concentrations, with a fluorescence fading time of 45 min.^[^ ^188^ ^]^

**Figure 25:**
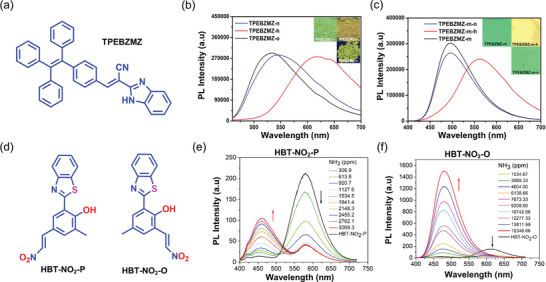
Chemical structures of a) TPEBZMZ, d) HBT‐NO_2_‐P, and HBT‐NO_2_‐O. b) Fluorescence spectra and image of TPEBZMZ‐O, original powder, TPEBZMZ‐h (HCl‐exposed powdered from TPEBZMZ‐O), and TPEBZMZ‐n (NH_3_‐exposed powdered from TPEBZMZ‐n). c) Fluorescence spectra and image of TPEBZMZ‐m, the original TPEBZMZ‐based nanofibrous film, TPEBZMZ‐m‐h (HCl‐exposed film from TPEBZMZ‐m), and TPEBZMZ‐m‐n (NH_3_‐exposed film from TPEBZMZ‐m‐h). e,f) Emission spectra of HBT‐NO_2_‐P and HBT‐NO_2_‐P with gradual addition of NH_3_. b,c) Adapted under the terms of the CC‐BY Creative Common Attribution 4.0 International License.^[^ ^191^ ^]^ Copyright 2021, Frontiers Media. e,f). Adapted with permission.^[^ ^192^ ^]^ Copyright 2022, Elsevier.

Stimuli‐responsive luminescent film sensors should possess high photostability, quantum yield, tunable composite variations, good durability, and reusability.^[^ ^189^, ^190^ ^]^ In 2021, Liu et al.^[^ ^191^ ^]^ synthesized a TPE derivative named TPEBZMZ ((Z)‐1‐(1H‐benzo[d]imidazol‐2‐yl)‐2‐(4‐(1,2,2‐triphenylvinyl)phenyl)ethen‐1‐amine) (**Figure** **25**), an AIE‐active stimuli‐sensitive probe, and fabricated a nanofibrous composite film TPEBZMZ‐m consisting of polylactic acid (PLA) and TPEBZMZ, yielding stimuli‐responsive capabilities. The benzimidazole unit in TPEBZMZ protonates in the presence of an acid, leading to a red‐shifted emission from green to yellow (Figure 25b). TPEBZMZ's original green emission was regained by exposing it to ammonia. TPEBZMZ‐m fiber has similar changes (Figure 25c).

By leveraging the concept of ESIPT, recently, in 2022 Zheng et al. synthesized AIE+ESIPT active benzothiazole derivatives‐based probes for sensitive amine detection and monitoring of the decomposition of urea by urease.^[^ ^192^ ^]^ ESIPT molecules give more Stokes shift to AIE‐active materials, which confers a more sensitive luminescence response.^[^ ^193^, ^194^ ^]^ In order to investigate the change in fluorescence spectra, ammonia was gradually added to HBT‐NO_2_‐P ((E)‐2‐(benzo[d]thiazol‐2‐yl)‐6‐methyl‐4‐(2‐nitrovinyl)phenol) and HBT‐NO_2_‐O ((E)‐2‐(benzo[d]thiazol‐2‐yl)‐4‐methyl‐6‐(2‐nitrovinyl)phenol, Figure 25d) in HEPES buffer solution. The intensity of the emission peak at 571 nm steadily decreases while the emission peak at 451 nm increases (Figure 25e). The trend observed in fluorescence variation of HBT‐NO_2_‐O is resembling that of HBT‐NO2‐P in ammonia (Figure 25f), although their relative intensities are significantly different. The detection limit of the HBT‐NO_2_‐P and HBT‐NO_2_‐O probes toward ammonia in the solution state was reported to be 226 and 13 ppm, respectively. The blue‐shift in the emission spectra in the presence of base might be due to hydrogen deprotonation from the phenolic hydroxyl moiety, which inhibits the ESIPT process. The film made by drop‐casting has a reduced sensitivity due to the suppression of the ESIPT process in the solid state, whereas the film fabricated by the electrospinning technique provides a large surface area and high porosity for enhanced sensitivity and reactivity. The electrospun nanofiber also provides sufficient space for ESIPT to occur, thus not affecting sensitivity in the solution state.^[^ ^192^ ^]^

### 2.6 AIE‐Active Polymeric Macrocycles Electrospun Nanofiber Composites

Polymers enjoy multiple advantages, e.g., they exhibit highly versatile mechanical properties (many with a good combination of ductility and toughness), ease in molding and shaping (ease of processability), rich chemical structures and topologies (e.g., linear, hyperbranched, star‐shaped, or ladder), which can be tuned to deliver specific functionalities.^[^ ^195^, ^196^ ^]^ AIE‐active polymers can potentially be deployed in various applications like explosive detection, polymer light‐emitting diode, and soft stimuli‐responsive materials.^[^ ^196^ ^]^ Modified AIE‐active polymers can be used for electrospinning of nanofibers by directly dissolving them in a compatible solvent, thus avoiding the need to start the process by mixing AIE material with an unmodified polymer. Bespoke fluorescent polymers can be designed and functionalized with ligands comprising AIEgens, or synthesized directly from AIE monomers before electrospinning. As exemplified below, fluorescent polymeric nanofibers can yield promising results in various biological and engineering fields.^[^ ^197^, ^198^ ^]^

**Figure 26:**
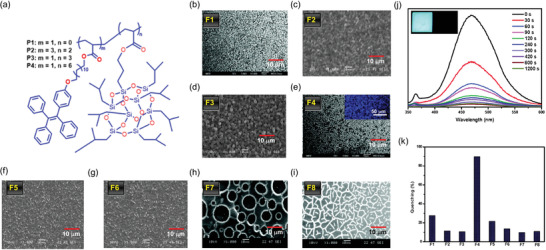
a) Chemical structures of the AIE‐active POSS polymer. SEM images of films of b) F1, c) F2, d) F3, e) F4 (inset taken under a fluorescence microscope with 337 nm excitation), f) F5, g) F6, h) F7, i) F8. The fabrication techniques are F1‐F6 by electrospinning, F7 by drop coating, F9 by spin coating. j) Fluorescence quenching of F4 upon exposure to DNT saturated vapor; insets show F4 before (left) and after exposure to DNT saturated vapor for 4 min under 365 nm UV irradiation at 25 °C. k) Emission quenching efficiency of all the fibers (F1–F8) in the presence of DNT vapors for 4 min at 25 °C. Adapted with permission.^[^ ^199^ ^]^ Copyright 2014, Royal Society of Chemistry.

Zhou et al.,^[^ ^199^ ^]^ reported in 2014 the synthesis of an AIE‐active polyhedral oligomeric silsesquioxane (POSS)‐based copolymers denoted as P1 to P4 (**Figure** **26**a). The resultant electrospun luminescent films exhibit a ninefold increase in sensitivity toward explosive vapors. The films were fabricated with varying concentrations of monomers via electrospinning (F1–F6), drop‐coating (F7), and spin‐coating (F8) methods. The SEM images of the F1 to F3 films show drastic morphological changes from short fibers with beads (Figure 26b), to small particles (Figure 26c), and to large particles (Figure 26d). Although the F4 film comprising P4 (POSS) shows a more uniform porous structure with a diameter of ≈300 nm (Figure 26e), it lacks a long fiber architecture that is commonly found in the electrospinning of a high‐molecular weight polymer. The SEM image of the F7 film shows a porous structure with large cavities (ranging from 2 to 10 µm) attributed to the evaporation of the solvents used in drop coating (Figure 26h). However, the F8 film shows an interlinked network with a cracked surface morphology (Figure 26i). The quenching effectiveness of porous film F4 was examined by exposing the F4 fiber to DNT (2,4‐dinitrotoluene) vapor for 4 min, during which time 90% of emission intensity was decreased (Figure 26j). By comparison, it was found that the fluorescence quenching effectiveness of the other co‐polymer fibers was less than that of the porous fiber F4 (Figure 26k). Compared to a dense film, the study shows that porous co‐polymer films lead to less dependence on the film thickness and improved sensitivity toward the detection of explosive vapors.^[^ ^199^ ^]^

**Figure 27:**
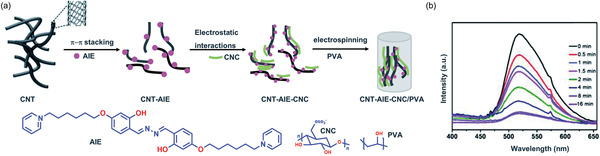
a) Schematic representation of the supramolecular ensemble of CNT‐AIE‐CNC as a filler for electrospinning to form the CNT‐AIE‐CNC/PVA composite. b) The emission intensity of CNT‐AIE‐CNC/PVA fiber in the presence of TNP (2,3,6‐trinitrophenol) vapor. Reproduced under the terms of the CC‐BY creative Common Attribution 3.0 International License .^[^ ^200^ ^]^ Copyright 2018, Royal Society of Chemistry.

Zhao et al.^[^ ^200^ ^]^ fabricated an electrospun nanofiber composite by leveraging the supramolecular approach to yield a strong and tough polymer fiber mat. The resultant nanofibrous composite consists of positively charged water‐soluble AIE material sandwiched between carbon nanotube (CNT) as a filler and carbon nanocrystals (CNC) as a reinforcing filler through *π*−*π* stacking and electrostatic interactions. This forms a water‐dispersible supramolecular ensemble of CNT‐AIE‐CNC, which further assembles with PVA through hydrogen bonding to yield the CNT‐AIE‐CNC/PVA composite (see **Figure** **27**a). The average diameter of the fiber is below ≈200 nm. Due to the hydrogen bonding between the polymer and fillers, the tensile strength, flexibility, elongation‐at‐break (ductility), Young's modulus, and toughness have markedly improved compared with the pristine PVA fiber.^[^ ^200^ ^]^ Adding TNP vapor quenched the fiber's emission intensity to 50% of its starting value in 2 min and to 70% in 8 min (Figure 27b). This study shows that rational design was enabled by a supramolecular approach, and the combination of different polymers could be employed for engineering mechanically resilient nanofibrous composites targeting fluorescent film sensors.

Thermosetting polymers are also known as thermosets. Because of their high degree of crosslinking, thermosets have better mechanical properties and chemical stability than thermoplastics. Thermosets are used in numerous applications, such as protective coatings, adhesives, composite engineering plastics, anticorrosive paints, construction equipment panels, electrical housing and components, insulators, heat shields, circuit breakers, agricultural feeding troughs, and motor components.^[^ ^201^, ^202^, ^203^ ^]^ However, the electrospun fibers of thermosetting polymers are difficult to fabricate due to the crosslinked nature of the polymeric chains.^[^ ^204^, ^205^ ^]^ When thermosetting polymer is combined with a curing agent, it starts to crosslink kinetically and with time and temperature, the viscosity of the resin increases. Due to the increase of viscosity with the extent of crosslinking, it is difficult to form a Taylor cone when exposed to an electric field in the electrospinning setup (Figure 3a inset), making electrospinning difficult to accomplish.^[^ ^201^, ^206^, ^207^ ^]^

**Figure 28:**
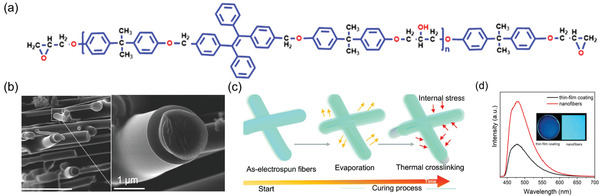
a) Chemical structures of the TPE‐modified thermosetting epoxy polymer. b) FESEM images of FNF‐2 and cross‐section showing the core–shell‐type fiber morphology. c) Proposed mechanism underlying the formation of core–shell morphology of FNF‐2, due to solvent evaporation and thermal crosslinking during curing process. d) Emission spectra of FNF‐2 as thin‐film coating versus electrospun nanofibers (inset: photoluminescence when excited under a 365 nm UV lamp). Adapted with permission.^[^ ^208^ ^]^ Copyright 2019, Royal Society of Chemistry.

In 2019, Li et al.^[^ ^208^ ^]^ synthesized a TPE‐modified epoxy resin (**Figure** **28**a), which emits strong PL emission due to the RIR mechanism. The modified epoxy resin is sensitive to external stimuli. The fluorescent nanofibers (FNFs) fabricated from the modified epoxy resin were highly photostable and can function as thermosensor and chemosensor probes. The study investigated factors including the role of solvents, pre‐crosslinking of epoxy resin, viscosity of the polymeric solution, and epoxy concentration for preparing the fluorescent thermosetting nanofibers. Figure 28b shows that the FNF‐2 fiber sample has a core–shell fiber morphology due to solvent evaporation and thermal crosslinking. The outer layer of the nanofiber dries quickly as the solvent evaporates, which generates an internal stress at the inner core of the nanofiber. During the thermal linking process, the inner core of the TPE‐modified epoxy resin is squeezed out to relieve the internal stress thus forming a core–shell type morphology (Figure 28c). The PLQY of the fibrous film is higher than the thin‐film coating, because the stretching of the polymers induces the restriction of the intramolecular rotation of the TPEs (Figure 28d). Furthermore, it was shown that the intramolecular rotation of the TPE rings can be leveraged to study the thermoset curing process of the electrospun epoxy nanofibers.^[^ ^208^ ^]^ In the following year, Zhao et al. adopted the above methodology for grafting functionalized AIE materials on electrospun nanofiber. This novel approach permitted real‐time study of the behavior of film sensors.^[^ ^209^ ^]^

**Figure 29:**
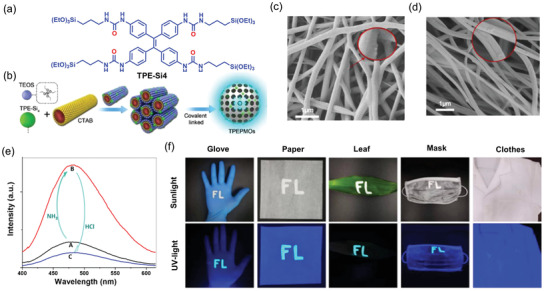
a) Chemical structure of TPE‐Si_4_. b) Scheme summarizing the preparation route of TPEPMOs. SEM images of the fibers of c) TPEPMO‐CF1 and d) TPEPMO‐CF2. e) The PL spectra of A) TPEPMO‐CF1 in the presence of B) NH_3_ and C) HCl. f) TPEPMO‐CF2 film sensors assembled onto a variety of materials, such as rubber glove, paper, leaf, facemask, and cloth. A polydopamine (PDA)‐mediated method was used for coating the surfaces of such varied materials. Adapted with permission.^[^ ^29^ ^]^ Copyright 2021, American Chemical Society.

In 2021, Gao et al.^[^ ^29^ ^]^ combined periodic mesoporous organosilica (PMOs) with a TPE derivative AIEgen to form the AIE‐active PMOs, termed TPEPMOs (**Figure** **29**a). Subsequently, TPEPMOs were embedded into poly(lactic‐*co*‐glycolic acid) and polyacrylonitrile fibers for fabricating acid/alkali optical sensors through the electrospinning technique. The SEM images of the TPEPMO‐CF1 and TPEPMO‐CF2 fibers (Figure 29b,c) show that TPEPMOs have been distributed uniformly through electrospinning; spherical particles on the surface of the nanofiber can be observed. The diameter of the individual TPEPMO‐CF1 fiber (320 nm) is higher than the TPEPMO‐CF2 fiber (250 nm). The fluorescence intensity of TPEPMO‐CF1 increases when exposed to ammonia vapor. TPEPMO‐CF2 has a stronger emission than TPEPMO‐CF1 due to the neutral precursor of the electrospinning solution. When fibers of TPEPMO‐CF1 were exposed to acid, the emission intensity declined, but it recovered when exposed to a base (Figure 29e). For evaluating potential practical applications, the study further shows that the TPEPMO‐CF fibers can be deposited onto various surfaces, such as gloves, masks, clothes, and papers (Figure 29f).^[^ ^29^ ^]^

The foregoing studies demonstrate the versatility of the concept of AIE‐active polymeric macrocycles for electrospinning multifunctional fibers. While there is a huge scope to design the specific chemistry of AIE‐polymer composites, precise control and tuning of the fiber architecture appears to be more challenging when working with thermosets. Further development in this field is warranted to gain a better handle of the entire process.

## 3 Summary and Perspectives

This review concerns a vibrant topic of research that is gaining a huge momentum, focusing on new materials innovations through merging of the concepts of AIEgens with electrospinning. The outcomes are striking, encompassing fine‐scale fluorescent fibers (micro‐ and nanofibers) with unconventional physical and chemical properties, targeting a vast range of technological applications. Herein, we have presented the core concepts of AIE materials and electrospinning, before discussing the most up‐to‐date reports pertaining to AIE‐based electrospun fibers (the first of which emerged only less than a decade ago), followed by detailed exemplars of the potential exploitations of fibrous thin films and porous membranes, and bespoke nanocomposites in many sectors. For example, fluorescent nanofibers incorporating AIEgens could be employed in optoelectronics, optical sensors of chemical and physical stimuli, drug delivery systems, as bioprobes and photothermal agents. Generally, the electrospun polymer fibers offer mechanical flexibility and stretchability, porous mesh architecture and scaffolding, and a high surface area with diverse morphology.^[^ ^210^, ^211^ ^]^ The results to date clearly demonstrate that, the scope for designing and tuning the AIE building blocks coupled with the base polymers are immense to achieve specific functions. Undoubtedly, there are challenges and opportunities in this nascent field, including the points we summarized below.

1) It will be prudent to investigate the precise AIE–polymer interaction controlling the photoefficiency of the electrospun fibers. It is generally suggested that electrospinning induces a physical stretch to the polymer matrix in the spinning process, inhibiting the intramolecular *π*−*π* interaction of AIEgen thereby enhancing the total radiative transition. However, direct experimental evidence is missing, which may be obtained by means of in situ or operando studies (e.g., imaging, scattering, diffraction measurements) at synchrotron and neutron facilities. Solid‐state NMR studies and vibrational terahertz spectroscopy may unravel the details of molecular rotor dynamics prevalent in AIEgens, and the specific rotor−polymer interactions found in electrospun fibers.
2) Computer simulations of AIE–polymer systems are not available to elucidate the fundamental mechanisms underlying structure–property relationships. Modeling common AIEgens (e.g., TPE and TPA derivatives) embedded in polymers could shed new light on the basic processes and help explain mechanisms responsible for the physicochemical behavior observed in experiments. The challenge may lie in the accurate theoretical representation of a complex molecular model incorporating weak interactions in a composite system comprising long‐chain polymers and bulky AIEgens.
3) The mechanical properties of AIE‐polymer systems have not been systematically studied, although the functions and performance of AIEgens greatly depend on the structural efficacy at the material interface. For instance, understanding mechanoluminescent composite fibers' elastic and plastic behavior is essential for practical implementations. Detailed studies on the time‐dependent mechanical response (e.g., creep and stress relaxation), structural durability, and fracture of electrospun films and membranes are also key for technological use. It is important to establish precisely how the mechanical behavior of the highly stretchable polymeric fibers can be harnessed to control the aggregation process via nanoscale confinement, which will pave the way to directing the structure and function of AIEgens confined in fibers.
4) AIE materials have emerged as an unconventional photothermal agent due to their large number of bulky molecular rotors and twisted structures. The composite of specific polymeric nanofiber comprising photothermal AIE‐active materials yield unusual properties that can be exploited for photodynamic applications, such as solar steam generation and in theranostics. Further work in this direction is warranted to improve the composites performance and long‐term material durability.
5) By understanding the structure–property relationship between the fiber and AIE materials, one can in principle rationally design and develop a fluorescent nanofiber with high external quantum efficiency and photostability. Nevertheless, detailed photophysical studies remain scarce, where fluorescence decay lifetime, excitation‐emission maps, and long‐term photostability/photobleaching behavior should be fully characterized. Local nanoanalytical techniques such as infrared nanospectroscopy (nanoFTIR),^[^ ^212^ ^]^ confocal Raman, and fluorescence lifetime imaging microscopy (FLIM) could reveal fine‐scale structure–property information that cannot be determined from bulk methods that give only an averaged value. It will also be important to study the role of defects^[^ ^213^ ^]^ on the performance of AIE‐active fibers.
6) The ability to design customized AIE materials and grafting them on the electrospun nanofibers gives a new strategy for constructing specific and real‐time fluorescent sensors that can be deployed in the solid state, without suffering from ACQ effects. Highly porous thin‐film sensors will be very efficient for vapor phase sensing of VOCs.
7) Research thus far has adopted TPE and TPA derivatives to fabricate AIE‐active electrospun nanofibers. Many other possible combinations of AIE‐active molecules and polymeric materials are yet to be explored. Novel properties to be discovered in AIE‐derived nanofibers include RTP, thermally activated delayed fluorescence, and NIR emissions, which will offer a unique platform for engineering stimuli‐responsive sensors and devices.
8) The concept of AIE@MOF holds considerable promise, as the first exemplar of the TPE@ZIF‐71 system^[^ ^182^ ^]^ has revealed exciting prospects for designing unusual photophysical and photochemical properties, realized by the encapsulation of an AIE guest in the highly versatile MOF host.^[^ ^14^ ^]^
9) The electrospinning technique has a limitation in that it requires polymers with a high molecular weight, essential for manufacturing good quality fibers. However, electrospinning of AIE‐based supramolecular systems (Section 2.6) could circumvent the need to work with high molecular weight polymers. Electrospun supramolecular fibers based on phospholipids, surfactants, crown ether derivatives, and cyclodextrins are scarce. These fibers face various hurdles, including limited mechanical stability, the rarity of stimuli‐sensitive luminous fibers derived from supramolecular systems, and the lengthy synthesis procedure for atypical supramolecular systems.^[^ ^214^, ^215^, ^216^ ^]^ When done right, AIE supramolecular systems could yield bright aggregate emission, a high photobleaching threshold, and a good signal‐to‐noise ratio sensor.^[^ ^55^ ^]^ We may overcome the foregoing restrictions by combining AIE supramolecular system with electrospinning to generate a new class of hierarchical materials with diversified architecture—ideal as a platform for biological systems.
10) Other exciting opportunities to explore are pH‐responsive electrospun fibers that may be utilized for target‐specific drug release, and could be fabricated by combining AIEgen with electrospun nanofibers. The hydrophobicity of the nanofibers and the effective optical features of the AIEgens could be utilized for a range of stimuli‐sensitive probes, such as surface pressure sensors, temperature‐responsive self‐cleaning materials, humidity‐responsive materials, protein‐responsive electrospun nanofibers, and chemo‐responsive fibers that will be attractive to the field of flexible electronics. Finally, it should be possible to engineer the polymer and AIEgens into a single strand of nanofiber acting as a system of multiple responsive ratiometric probes, which would be beneficial in biomedical applications for target‐specific therapeutic research and in soft robotics for mimicking bimolecular machines.

To conclude, this review demonstrates that the AIE research in recent decades has rapidly propagated into various fields, encompassing physics, chemistry, materials science, optics and electronics, engineering science, and biomedical sciences. The diversity of the AIE‐derived composite materials is immense, and there are numerous opportunities to engineer innovative material systems. Rational design of bespoke AIEgen materials coupled with electrospinning methodologies could revolutionize the way future materials are being designed and engineered toward specific functions (Figure 2), overcoming the current limitations of conventional PL materials that suffer from ACQ effects (Figure 1). To this end, it is paramount to establish a deeper understanding of the composites system comprising the aggregated AIEgens embedded in fiber micro/nanoenvironment, focusing on structure–property relationships, polymer–AIEgen interactions, and the role of fiber architecture. Further insights into the effects of aggregation on the molecular properties of electrospun fibers could leapfrog the innovation of porous functional materials destined for advanced technologies.

## Conflict of Interest

The authors declare no conflict of interest.
